# Tissue-level alveolar epithelium model for recapitulating SARS-CoV-2 infection and cellular plasticity

**DOI:** 10.1038/s42003-022-03026-3

**Published:** 2022-01-19

**Authors:** Jia-Wei Yang, Yu-Rou Lin, Ying-Ling Chu, Johnson H. Y. Chung, Huai-En Lu, Guan-Yu Chen

**Affiliations:** 1 Department of Electrical and Computer Engineering, College of Electrical and Computer Engineering, National Yang Ming Chiao Tung University, Hsinchu, Taiwan; 2 Institute of Biomedical Engineering, College of Electrical and Computer Engineering, National Yang Ming Chiao Tung University, Hsinchu, Taiwan; 3 ARC Centre of Excellence for Electromaterials Science, Intelligent Polymer Research Institute, University of Wollongong, Wollongong, NSW Australia; 4 Bioresource Collection and Research Center, Food Industry Research and Development Institute, Hsinchu, Taiwan; 5 Department of Biological Science and Technology, National Yang Ming Chiao Tung University, Hsinchu, Taiwan

**Keywords:** Respiratory system models, Viral infection

## Abstract

Pulmonary sequelae following COVID-19 pneumonia have been emerging as a challenge; however, suitable cell sources for studying COVID-19 mechanisms and therapeutics are currently lacking. In this paper, we present a standardized primary alveolar cell culture method for establishing a human alveolar epithelium model that can recapitulate viral infection and cellular plasticity. The alveolar model is infected with a SARS-CoV-2 pseudovirus, and the clinically relevant features of the viral entry into the alveolar type-I/II cells, cytokine production activation, and pulmonary surfactant destruction are reproduced. For this damaged alveolar model, we find that the inhibition of Wnt signaling via XAV939 substantially improves alveolar repair function and prevents subsequent pulmonary fibrosis. Thus, the proposed alveolar cell culture strategy exhibits potential for the identification of pathogenesis and therapeutics in basic and translational research.

## Abstract (web summary)

Yang et al. present an alveolar cell culture method that utilizes primary human alveolar epithelial cells, culturing cells in a small-molecule cocktail supplemented media. The authors show that the longevity of the primary cells culture can be expanded, and study human alveolar development, infection, injury repair, while demonstrating its use for SARS-CoV-2 infection.

## Introduction

Recovered COVID-19 patients encounter many post-viral complications, which include abnormal pulmonary function (25%)^1^, abnormal pulmonary diffusion (26%)^2^, and severe pulmonary fibrosis (7–8%)^3^. Although previous studies have investigated the mechanism of the SARS-CoV-2 infection^4,5^, the identification of pathogenesis and effective therapeutic strategies remains a challenge because of limited cell sources^6,7^. Therefore, the development of a standardized human lung-cell culture method has become imperative in the post-COVID-19 era, which provides an important opportunity to create a functional model of the human lung for studying the sequelae of COVID-19 pneumonia.

Primary human pulmonary alveolar epithelial cells (HPAEpiCs) composed of alveolar type-I (AT1) and type-II (AT2) cells are the major cells infected by SARS-CoV-2^8^. Unfortunately, there is a lack of culture methods for maintaining the HPAEpiC phenotype for more than 14 days or greater than 3 passages^9,10^. Thus, HPAEpiC-based models are restricted to studying the viral infection and inflammatory response within a short-term period; they fail to investigate the biological mechanisms of alveolar damage and repair adequately. Thus far, the existing literature extensively considered AT2 cells as progenitor cells that play crucial roles in alveolar regeneration and repair^11,12^; however, several contrasting studies revealed that AT1 cells have the potential to promote alveolar tissue renewal and regeneration^13–15^. Further, a recent study has determined that AT1 cells primarily function not only to provide gaseous exchange, but also to serves as critical signaling hubs for alveolar niche remodeling^16^. Although many uncertainties exist in terms of the interaction between AT1 and AT2 cells, they are indeed indispensable for alveolar regeneration and repair. In addition, both AT1 and AT2 cells are involved in the regeneration of alveolar epithelium after the SARS-CoV-2 infection^17^. Thus, a unique alveolar model that recapitulates the specific functions of AT1 and AT2 cells is crucial for providing important insights into therapeutic strategies and pathogenic mechanisms that follow an alveolar injury.

In this study, we aim to develop a standardized method for long-term HPAEpiC culture that can enable the establishment of a tissue-level alveolar model to recapitulate the air–liquid interface (ALI) environment, alveolar epithelial barrier integrity, pulmonary surfactant production, and cellular plasticity. During the modeling of the human alveolar epithelium, we found that the initiation of the ALI condition after 7 and 14 days of submerged (Sub) conditions ensures a 10% fraction of AT2 cells in the alveolar model, which is comparable to that in human alveoli^18^. We observed that the established alveolar model reproduces a heterogeneous expression of angiotensin-converting enzyme 2 (ACE2) and transmembrane protease, serine 2 (TMPRSS2)^19^, and the clinical characteristics of the SARS-CoV-2 infection in human alveoli^20^. We demonstrated that this previously uncharacterized alveolar model successfully recapitulates the plastic fates of AT1 and AT2 cells during alveolar homeostasis and post-injury repair. Wound healing in damaged alveolar epithelium was enhanced, fibroblast activation by the active promotion of AT1 and AT2 cell interactions was prevented, and the cellular phenotype was maintained by regulating Wnt signaling. These findings highlight the emergence of a revolutionary HPAEpiC culture strategy, which is a valuable in vitro alveolar model to study human alveolar development, disease, repair, and regeneration.

## Results

### Customized culture medium for in vitro growth of HPAEpiCs

**Fig. 1:**
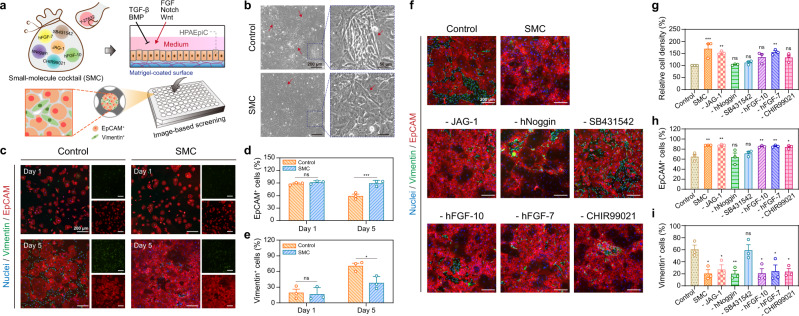
Development of customized HPAEpiC cell culture medium. **a** Schematic of screening process for HPAEpiC culture medium development. HPAEpiCs were cultured with various small molecules to mediate growth; a high-content imaging system was used to identify the EpCAM^+^ and Vimentin^+^ cell populations. **b** Representative images of HPAEpiCs cultured with control and SMC media. The arrowhead indicates elongated spindle-shaped cells (mesenchymal-like cells). **c** The HPAEpiCs were stained with Vimentin (green) and EpCAM (red) antibodies, which are specific markers for mesenchymal and epithelial cells, respectively, after 1 and 5 days of culturing. Quantification of EpCAM^+^ (**d**) and Vimentin^+^ (**e**) cell populations. The SMC medium supports epithelial cell phenotype maintenance after 5 days of culturing. **f** The HPAEpiCs were stained with Vimentin (green) and EpCAM (red) antibodies following culturing in various small-molecule media for 5 days. **g** Cell-density quantification after 5 days of culturing. The SMC, -JAG-1, and -hFGF-7 media have higher cell growth density than the control medium. Quantification of EpCAM^+^ (**h**) and Vimentin^+^ (**i**) cell populations after 5 days of culturing. SB431542 plays a crucial role in preventing epithelial to mesenchymal transition. Data and error bars represent mean ± SEM from three separate experiments. Two-way (**c**, **d**) and one-way ANOVA tests (**g**–**i**). *p  <  0.05; **p  <  0.01; ***p  <  0.001; ns non-significant.

We defined a small-molecule cocktail (SMC) medium, which was supplemented with Jagged-1 peptide (JAG-1), recombinant human Noggin protein (hNoggin), SB431542, recombinant human fibroblast growth factor (hFGF)-10 protein, hFGF-7, CHIR99021, and Y-27632, as an initial step in designing a standard method for long-term 2D culture of HPAEpiCs to regulate the growth behavior through the corresponding signaling pathways (Supplementary Table 1)^21^. We confirmed the morphological changes to identify the expression of the epithelial cell adhesion molecule (EpCAM) and Vimentin—specific markers of epithelial and mesenchymal cells, respectively—to determine whether the SMC medium supports in vitro growth of HPAEpiCs (Fig. 1a). Given the limited cell resources and numbers, an automated high-content imaging system was used to acquire the immunofluorescence images of all regional cells and perform unbiased quantitative analysis by fluorescent cell counting, which allowed the efficient screening of diverse supplements at the maximum possible sample size^22^.

HPAEpiCs isolated from the human lung tissue were obtained from a commercial supplier (ScienCell Research Laboratories) and authenticated by the manufacturer. As mentioned in the literature^21^, 98% of the HPAEpiCs expressed EpCAM and 42% of the EpCAM^+^ cells expressed the human AEC2 specific marker. In the experiments, HPAEpiCs were seeded on Matrigel-coated 96-well plates and incubated with the SMC medium. After 5 days of culturing, almost all cells exhibited epithelial morphology (cobblestone-like), which suggests that the SMC medium can inhibit transformation to an elongated spindle-shaped morphology (Fig. 1b). Further, we determined that almost all cells in the SMC medium were EpCAM^+^ cells, whereas a large number of Vimentin^+^ cells were present in the supplier medium (control medium) (Fig. 1c). The statistical analysis revealed the ratio of EpCAM^+^ cells to Vimentin^+^ cells in the SMC medium maintained for more than 2 (89% EpCAM^+^ cells; 38% Vimentin cells), whereas it was only ~0.8 (58% EpCAM^+^ cells; 70% Vimentin cells) in the control medium (Fig. 1d, e). The day 1 data indicated that ~17.5% of Vimentin^+^ cells were observed in the control and SMC medium; however, almost no cells co-expressed EpCAM, which implies a few epithelial–mesenchymal transition (EMT) cells are present in the original cryopreserved at P0^23^. However, the EpCAM^+^ and Vimentin^+^/EpCAM^+^ cells were maintained in the SMC condition, whereas a considerable number of Vimentin^+^/EpCAM^−^ mesenchymal cells were observed in the control group. This suggests that the SMC medium can support the growth and proliferation of HPAEpiCs while maintaining the cell phenotype.

We removed single additives, i.e., –JAG-1, –hNoggin, –SB431542, –hFGF-10, –hFGF-7, –CHIR99021, and –Y-27632, to evaluate the necessity of various supplements in the SMC medium (Supplementary Table 2). We performed the same general and statistical analyses as described above after the HPAEpiCs were cultured under different conditions for 5 days (Fig. 1f). First, the relative cell density showed that the removal of hNoggin, SB431542, hFGF-10, and CHIR99021 altered the HPAEpiC attachment and proliferation (Fig. 1g). Second, the removal of hNoggin and SB431542 affected the EpCAM^+^ cell population percentage (Fig. 1h). Third, SB431542 played a key role in preventing HPAEpiC transformation from EpCAM^+^ to Vimentin^+^ cells (Fig. 1i). These results provide important insights into support provided by the SMC medium for the in vitro culture of the HPAEpiCs, which emphasizes the realization of the stable culture of HPAEpiCs.

### Effective expansion of HPAEpiCs by maintaining phenotype

**Fig. 2:**
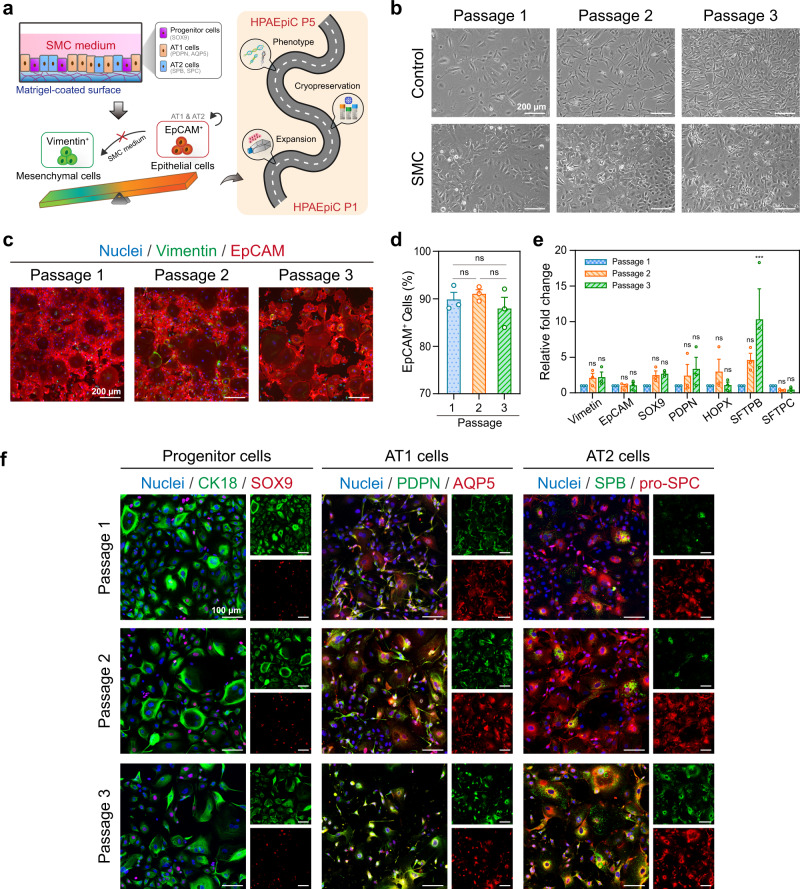
SMC medium support of HPAEpiC growth and expansion in serial passages. **a** Schematic of experiment showing the SMC medium support of long-term HPAEpiC passage culture. Experiments were devised to determine whether the HPAEpiC phenotype was maintained after expansion and cryopreservation. **b** Bright-field microscope images showing changes in HPAEpiC morphology after the serial passage. In the control medium, the Passage-2 cells were almost elongated spindle-shaped (mesenchymal-like cells), whereas the SMC medium maintained a cobblestone epithelial morphology from passages 1 to 3. **c** When serial passaging was performed in the SMC medium, the HPAEpiCs were stained with Vimentin (green) and EpCAM (red) antibodies. **d** Quantification of EpCAM^+^ cell populations. Data showed that the SMC medium supports maintenance of the HPAEpiC epithelial cell phenotype between Passages 1 and 3. **e** mRNA expression of *Vimentin* (mesenchymal cells), *EpCAM* (epithelial cells), *SOX9* (alveolar progenitor cells), *PDPN* and *HOPX* (AT1 cells), as well as *SFTPB* and *SFTPC* (AT2 cells). **f** Immunostaining of CK18 and SOX9 (alveolar progenitor cells), PDPN and AQP5 (AT1 cells), as well as SPB and pro-SPC (AT2 cells). From **d**–**f**, the SMC medium supports the maintenance of the alveolar progenitor, AT1, and AT2 cell phenotypes of the HPAEpiCs between Passages 1 and 3. Data and error bars represent mean ± SEM from three separate experiments. One-way ANOVA test (**d**, **e**). ***p  <  0.001; ns non-significant.

We attempted to determine whether the SMC medium supports serial HPAEpiC passaging and expansion (Fig. 2a). We observed that, after three passages of HPAEpiCs in the control medium, almost all cells transformed to elongated spindle-shaped and Vimentin^+^ cells; this is like the human lung fibroblast cell line HFL1 (Supplementary Fig. 1a–c). These results are consistent with previous findings, and the emphasize the challenge of the long-term HPAEpiC culture^24–26^. However, HPAEpiCs maintained the epithelial morphology and EpCAM expression over three passages in the SMC medium (Fig. 2b, c). More surprisingly, statistical analysis confirmed that 90% of the EpCAM^+^ cells were present in HPAEpiC Passages 1 to 3 (Fig. 2d). We performed real-time quantitative reverse transcription–polymerase chain reaction (qRT-PCR) analysis to examine the gene expression of the passaged HPAEpiCs, which includes *Vimentin* (mesenchymal cells), *EpCAM* (epithelial cells), *sex-determining region Y–box transcription factor 9* (*SOX9*; alveolar progenitor cells), *podoplanin* (*PDPN*; AT1 cells) and *HOP homeobox* (*HOPX*; AT1 cells), and surfactant proteins B and C (*SFTPB* and *SFTPC*; AT2 cells) (Fig. 2e). Significant changes were not observed for most gene expression levels, with only the *SFTPB* gene (mature AT2 cells) exhibiting a significant increase after three passages. The increase in *SFTPB* expression explains the transition from immature to mature cells because all HPAEpiCs used in this analysis were derived from fetal lungs (~20 weeks); however, human alveolus maturation occurs from embryonic day 252 to 3 years^27,28^. Further, immunofluorescence staining revealed that the expression of the associated proteins, i.e., the cytokeratin 18 (CK18; epithelial cells) and SOX9 (progenitor cells), PDPN and aquaporin 5 (AQP5; AT1 cells), and surfactant protein B (SPB) and pro-surfactant protein C (pro-SPC; AT2 cells), were continuously maintained for up to five passages (Fig. 2f and Supplementary Fig. 1d, e). Almost all cells expressed both AT1 and AT2 markers, which is a characteristic of immature alveolar epithelial cells during human lung development^29,30^. We demonstrated that the cryopreservation of the passaged HPAEpiCs maintained the cell phenotype (Supplementary Fig. 2). Therefore, these results indicated that the SMC medium adequately supports HPAEpiC growth, expansion, and preservation. These results indicated a feasible method for the long-term culture of HPAEpiCs in conventional culture dishes and multi-well plates; this is a breakthrough in the large-scale expansion culture of human alveolar cells.

### Reconstitution of functional human lung alveolar in vitro model

**Fig. 3:**
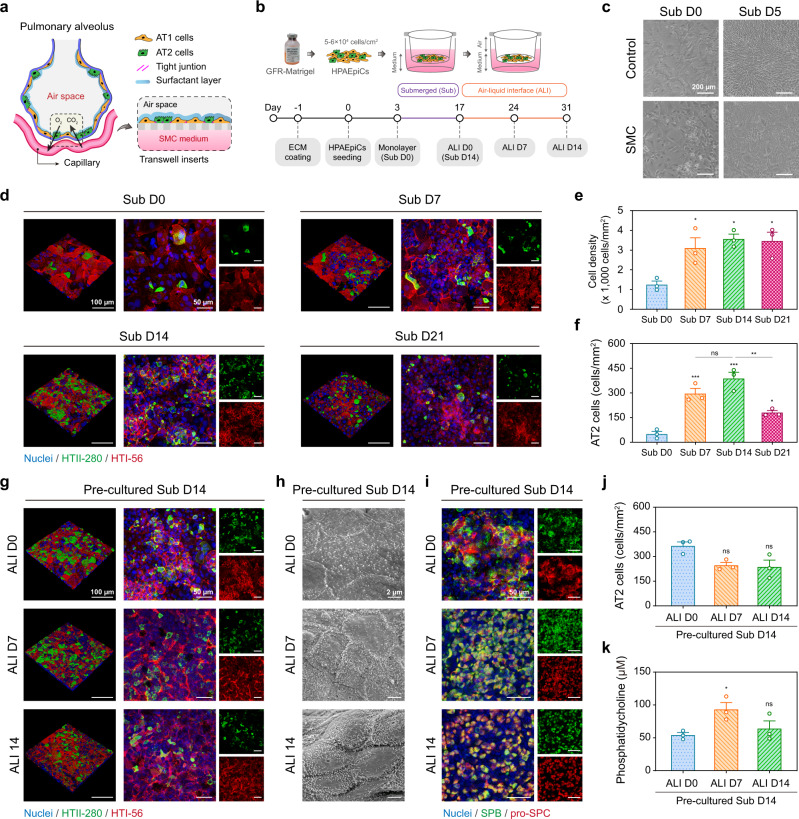
Reconstitution of functional human alveolar model with ALI. **a** Schematic of alveolar model containing AT1 cells, AT2 cells, epithelium integrity, and surfactant layer. **b** Timeline flowchart showing HPAEpiC culture steps during modeling process. The HPAEpiCs were submerged (Sub) cultured for 14 days, and then, ALI cultured for 14 days. **c** Bright-field microscope images showing changes in HPAEpiC morphology during the modeling process. In the control medium, cells were almost elongated spindle-shaped after 5 days of Sub-culturing, whereas the SMC medium maintained an epithelial morphology. **d** During Sub-culturing, HPAEpiCs were stained with HTII-280 (green) and HTI-56 (red) antibodies, which are specific markers for AT2 and AT1 cells, respectively. **e** Cell-density quantification for 21 days of sub-culturing. The HPAEpiCs proliferated significantly in the first 7 days before the proliferation trend gradually stabilized. **f** Quantification of HTII-280^+^ cell (AT2 cells) populations for 21 days of Sub-culturing. The AT2 cell number reached a maximum at 14 days of culturing and then declined. **g**–**k** After 14 days of Sub pre-culturing, the HPAEpiCs initiated the ALI culture condition for 14 days of culturing. During ALI culturing, HPAEpiCs were stained with HTII-280 (green) and HTI-56 (red) antibodies (**g**), and cell microstructures were observed using a scanning electron microscope (**h**). In addition, the HPAEpiCs were stained with SPB (green) and pro-SPC (red) antibodies (**i**), both of which are specific markers for AT2 cells and alveolar surfactant protein. Quantification of HTII-280^+^ cell (AT2 cell) populations (**j**) and PC concentration (**k**) for 14 days of ALI culturing. From **g**–**k**, ALI culturing enhances the epithelium integrity and surfactant production. Data and error bars represent mean ± SEM from three separate experiments. One-way ANOVA test (**e**, **f**, **j**) and Student’s *t* test (**k**). *p < 0.05; **p < 0.01; ***p < 0.001; ns non-significant.

There is no doubt that the ALI culture provides physiologically relevant advantages for lung models^31,32^ such as epithelial cell surface polarization and pulmonary surfactant production (Fig. 3a). We first culture HPAEpiCs in Transwell inserts for 7, 14, and 21 days under submerged (Sub) and ALI conditions to optimize the timeline for establishing a human alveolar model; these samples are labeled Sub D7, D14, D21, and ALI D7, D14, D21, respectively (Fig. 3b and Supplementary Fig. 3a). Compared with the control medium, the SMC medium maintains the HPAEpiC morphology (Fig. 3c). Interestingly, we found higher numbers of human type-II (HTII)-280^+^ AT2 cells under continuous Sub conditions compared to continuous ALI conditions, which suggests that AT2-cell proliferation efficiency depends on the culture environment (Supplementary Fig. 3b–d). Further investigations revealed that active HTII-280^+^ AT2 cell growth (i.e., between Sub D7 and Sub D14) occurred following the formation of monolayer human type-I (HTI)-56^+^ AT1 cells; proliferating AT2 cells were located on the apical surface of the cell layer (Fig. 3d, e), which suggests that the formation of AT1 cell monolayers is an important niche for AT2 cell proliferation. This supports physiological findings that AT1 cells predominantly cover the alveolar surface as a barrier structure whereas AT2 cells exist in the alveolar corner^33^. Statistical analysis showed that proliferating AT2 cells increased to 10% of the total population at Sub D7 and Sub D14, which is close to the fraction for human alveoli (Fig. 3f)^18^. Therefore, initiating the ALI condition after a period under the Sub condition can ensure a certain number of AT2 cells in the alveolar model.

Subsequently, we implemented the ALI culture condition for 0, 7, and 14 days with a precultured Sub D14 alveolar model (precultured Sub D14 + ALI D0, D7, and D14) to determine the indispensability of the ALI condition. Under this condition, we observed that the alveolar model formed a more compact monolayer and maintained the HTII-280^+^ cell population within the tissue level (Fig. 3g, j and Supplementary Fig. 4). Scanning electron images revealed that the ALI condition reconstructed the alveolar epithelium integrity by promoting intercellular junction maturation and microvilli growth (up to 0.4–1 μm) (Fig. 3h and Supplementary Fig. 5). We found the abundant expression of SPB and pro-SPC under the ALI condition, as shown in Fig. 3i, which suggests the presence of AT2 cells and alveolar surfactant protein. Interestingly, the surfactant protein distribution changed from the cytoplasm to the cell membrane, and it may be attributed to cell surface polarization. Finally, the phosphatidylcholine (PC) concentration, where PC comprises 60% of the pulmonary surfactant in human alveoli^34^, increased significantly by 1.7 times under the precultured Sub D14 + ALI D7 condition (Fig. 3k). In the current study, our alveolar model could be compared to normal human alveoli tissue in vivo, recapitulating 10% of the AT2 cells and the pulmonary surfactant production, alveolar epithelial integrity, and microvilli structures (Supplementary Table 3). This optimized ALI condition could help establish large-scale alveolar models with different passages of HPAEpiCs (Supplementary Fig. 6). These findings suggest that physiological-level alveolar epithelial models can be recapitulated when HPAEpiCs are precultured in the Sub condition for 7–14 days and then incubated in the ALI condition for 7–14 days.

### SARS-CoV-2 productive infection of human alveolar model

**Fig. 4:**
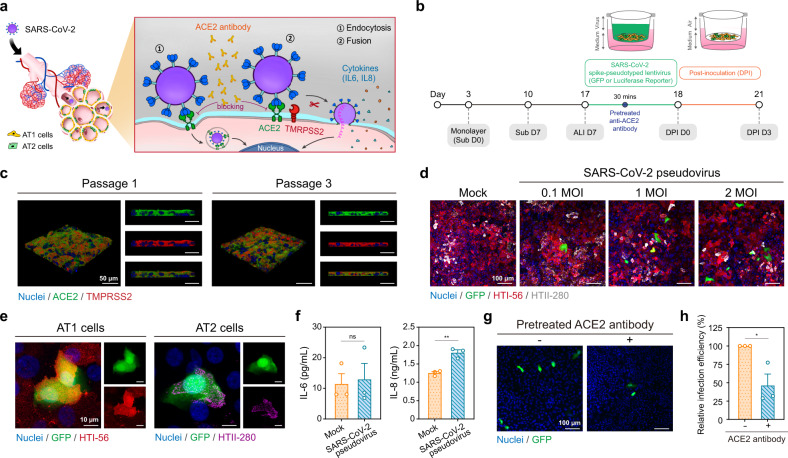
SARS-CoV-2 infection in human alveolar model. **a** Schematic of experiment in which HPAEpiCs were infected with SARS-CoV-2 pseudovirus. Experiments were devised to determine whether the AT1 and AT2 cells in our model could be infected by the virus, whether the model could serve as a preclinical testing platform for drug strategy development. **b** Timeline flowchart showing HPAEpiC culture steps during SARS-CoV-2 pseudovirus infection process. For virus infection, the human alveolar model was established under Sub-culturing for 7 days and ALI culturing for 7 days. **c** After the human alveolar model was established, the HPAEpiCs were stained with ACE2 (green) and TMPRSS2 (red) antibodies, both of which are key proteins for SARS-CoV-2 to enter target cells. **d**, **e** After 3 days of SARS-CoV-2 pseudovirus infection (GFP reporter, MOI = 0.1, 1, and 2), HPAEpiCs were stained with HTI-56 (red) and HTII-280 (purple) antibodies, and the GFP fluorescence signal was observed (**d**). High-magnification images showing AT1 and AT2 cells express GFP fluorescence, which indicate that both cell types were infected with SARS-CoV-2 pseudovirus (**e**). **f** After 3 days of SARS-CoV-2 pseudovirus infection (MOI = 2), proinflammatory cytokine IL-6 and IL-8 concentrations produced by the HPAEpiCs were confirmed. IL-8 was released at a significant level when the HPAEpiCs were infected with the SARS-CoV-2 pseudovirus. **g**, **h** Before virus infection, the HPAEpiCs were incubated with anti-ACE2 antibody for 30 min to block ACE2 receptors. After 3 days of SARS-CoV-2 pseudovirus infection (GFP reporter, MOI = 2) of the ACE2-blocked alveolar model, the GFP fluorescence signal was observed (**g**) and the infection efficiency was quantified (**h**). The anti-ACE2 antibody-pretreated model reduced the infection efficiency by half. Data and error bars represent mean ± SEM from three separate experiments. Student’s *t* test (**f**, **h**). *p < 0.05; **p < 0.01; ns non-significant.

We devised viral infection and antibody blocking experiments with a SARS-CoV-2 pseudovirus (green fluorescent protein (GFP) and luciferase reporter genes) to assess the effect of COVID-19 on the alveolar model (Fig. 4a). We adopted a time-optimized alveolar model cultured under the precultured Sub D7 + ALI D7 condition to model the SARS-CoV-2 infection (Fig. 4b). Initially, we examined ACE2 and TMPRSS2 in an alveolar model because they are the entry receptor and major priming enzyme for the SARS-CoV-2 infection, respectively. Interestingly, the ALI condition stimulated the production and maturation of both ACE2 and TMPRSS2, which are co-localized on the cell surface (this is consistent with human alveoli) (Supplementary Fig. 7). We proved that the proposed HPAEpiC culture strategy can provide a large-scale alveolar model expressing ACE2 and TMPRSS2 for COVID-19 research (Fig. 4c and Supplementary Fig. 8).

For the viral infection, we attempted to replace the culture medium from the SMC with the control medium in the created model to prevent interference from culture media supplements. The immunofluorescence of the HTI-56^+^ AT1 cells and HTII-280^+^ AT2 cells indicated that the control medium could maintain the alveolar model for at least 3 days after SMC replacement (Supplementary Fig. 9). We confirmed the binding interaction between the SARS-CoV-2 spike protein and the alveolar model via western blot analysis (Supplementary Fig. 10). After 3 days of post-infection with the SARS-CoV-2 pseudovirus, fluorescent images showed that the number of GFP^+^ cells depended on the SARS-CoV-2 pseudovirus concentration (multiplicity of infection (MOI) = 0.1, 1, and 2) (Fig. 4d). Both the AT1 and AT2 cells were infected (Fig. 4e and Supplementary Fig. 11), which mirrors histopathological findings from autopsies^20^. In addition, we found that more Ki-67^+^ proliferating cells were observed in the infected model with the response of proinflammatory cytokine interleukin 8 (IL-8) (Fig. 4f and Supplementary Fig. 12a). The analysis of all types of alveolar surfactant proteins (SPA, SPB, pro-SPC, and SPD) indicated that the SPA expression decreased after the viral infection (Supplementary Fig. 12b, c). Although the SARS-CoV-2 pseudovirus has limitations in terms of the innate antiviral response to the SARS-CoV-2 infection, the findings indicate the potential of the proposed alveolar model to further study the physiological response.

Next, we examined whether the alveolar model could be used as a testing platform for COVID-19 drug development. When ACE2 receptors on the cell surface were blocked, SARS-CoV-2 failed to enter the cells^35^. Thus, we performed pre-treatment with an anti-ACE2 antibody before the pseudovirus infection to mimic an alveolar model that lacks ACE2 activity. After the pseudovirus infection, the fluorescence images showed that the number of GFP^+^ cells greatly decrease in the ACE2-pretreated alveolar model (Fig. 4g). Further statistical analysis reveals that the viral infection efficiency is reduced by half (Fig. 4h). Thus, our results collectively demonstrated that the established alveolar model not only reproduces the SARS-CoV-2 infection, but also provides the potential support required for drug development.

### Effect of Wnt signaling on alveolar repair and intervention of pulmonary fibrosis

**Fig. 5:**
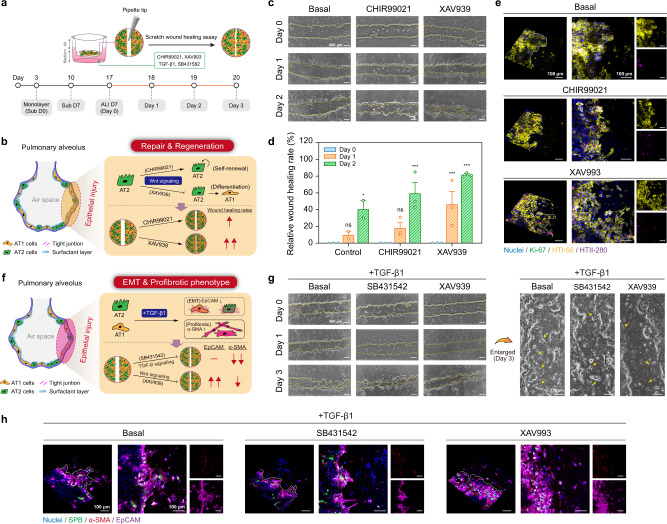
HPAEpiC repair response in damaged human alveolar model. **a** Timeline flowchart showing HPAEpiC culture steps during wound healing process under ALI culturing condition. The HPAEpiCs were maintained under Sub-culturing for 7 days and ALI culturing for 7 days to create a damaged human alveolar model; then, a 200-μl pipette tip was used to scratch the wound area. **b** Schematic of experiment on HPAEpiC repair and regeneration after injury. Both Wnt signaling activation (CHIR99021) and inhibition (XAV939) improved wound healing efficiency. **c** Bright-field microscope images showing changes in wound healing process after treatment with CHIR99021 and XAV939. The dotted line represents the wound healing boundary. **d** Quantification of wound healing rate during 2 days post-scratching. The wound healing ability was in the order of XAV939 > CHIR99021 > control. **e** After 2 days of treatment, the HPAEpiCs were stained with Ki-67 (green), HTI-56 (yellow), and HTII-280 (purple) antibodies. The white dotted line represents the wound healing boundary, and it shows a certain number of HTI-56^+^ (AT1 cells) and HTII-280^+^ cells (AT2 cells), as well as a few Ki-67^+^ cells (proliferating cells) around the wound healing area. **f** Schematic of experiment on HPAEpiC repair and regeneration during TGF-β1 activated wound healing process. SB431542 (a TGF-β1 inhibitor) and XAV939 both reduced fibroblast activation. Importantly, XAV939 maintained the epithelial cell phenotype. **g** Bright-field microscope images showing changes in TGF-β1 activated wound healing process after treatment with SB431542 and XAV939. The dotted line represents the wound healing boundary, and the arrowhead indicates elongated spindle-shaped cells (mesenchymal-like cells). **h** After 3 days of treatment, the HPAEpiCs were stained with SPB (green), α-SMA (red), and EpCAM (purple) antibodies. The dotted line represents the wound healing boundary and shows a certain number of α-SMA^+^ cells (activated fibroblasts) around the wound healing area. In addition, XAV939 was observed to have more EpCAM expression. Data and error bars represent mean ± SEM from three separate experiments. Two-way ANOVA test (**d**). *p < 0.05; ***p < 0.001; ns non-significant.

The established alveolar model was treated with lipopolysaccharide (LPS) to demonstrate physiological changes in barrier integrity after host–pathogen interactions because only laboratories with a biosafety level 3 are allowed to handle the wild-type SARS-CoV-2 virus (Supplementary Fig. 13). We adopted the common mechanical scratching method to create a wound area in the alveolar model to further investigate the regeneration response of the damaged alveoli (Fig. 5a). We then aimed to regulate Wnt signaling activity changes in the damaged alveolar model in response to AT2-cell self-renewal and differentiation (Fig. 5b)^36^. We found that both the activation (CHIR99021) and inhibition (XAV939) of the Wnt signaling promoted the wound healing process (Fig. 5c). Based on the calculated wound area, XAV939 exhibited the highest wound healing rate (almost 82%) after 2 days of post-scratching, whereas CHIR99021 and the control group exhibited rates of 60% and 41%, respectively (Fig. 5d). We found that the HTII-280^+^ AT2 cells were concentrated at the boundary of the wound healing area in the damaged alveolar model treated with CHIR99021 and XAV939; this implies that the positive and negative regulation of Wnt signaling plays a key role in the alveolar epithelial regeneration of AT2 cells toward the wound area (Fig. 5e). Although previous studies reported that the inhibition of Wnt signaling promotes the differentiation of AT2 cells into AT1 cells^36,37^, a similar number of HTII-280^+^ AT2 cells were observed in the wound healing model compared to that in the Wnt signaling activation group. These findings suggest that alveolar regeneration can primarily be attributed to AT1–AT2 cell interactions such as bidirectional transdifferentiation of the AT1 and AT2 cells, rather than to the AT2 cells dominating the repair process;^36^ this supports the recent findings^15,16^.

Next, we attempt to determine whether XAV939 can prevent the development of pulmonary fibrosis after alveolar injury since this is one of the main sequelae of COVID-19 pneumonia. We add a transforming growth factor (TGF)-β1 to the damaged alveolar model to induce EMT and fibroblast–myofibroblast transdifferentiation to simulate a state of activated fibrosis (Fig. 5f). Following treatment with SB431512 (a TGF-β1 signaling inhibitor) and XAV939, we observed a modest reduction in the elongated spindle-shaped morphology, which is the main feature of mesenchymal cells and myofibroblasts (Fig. 5g). As shown in Fig. 5h, SPB^+^ AT2 cells are concentrated near the boundary of the wound healing area in the SB431512 and XAV939 treatment groups, which suggests that the inhibition of TGF-β1 and Wnt signaling under TGF-β1-induced fibrotic conditions mediate the progression of alveolar epithelium repair toward normal regenerative behavior, as indicated in the above experiments. Further, only XAV939 maintains the EpCAM expression, which implies the inhibition of Wnt signaling not only reduces the appearance of fibroblasts, but also effectively maintains the phenotype of alveolar epithelial cells. These findings provide important insights into the role of the Wnt signaling pathway in the transdifferentiation of HPAEpiC into fibroblast and α-smooth muscle actin (α-SMA)^+^ cells, which offers therapeutic possibilities for pulmonary fibrosis. We determined that the ALI-condition model is necessary for the in vitro study of alveolar repair because under the Sub condition, the damaged alveolar model almost completely heals within a day (Supplementary Fig. 14). Thus, our alveolar model not only recapitulates the physiological alveolar repair response, but also allows the dissection of the mechanism for disease treatment.

## Discussion

Human-relevant models are urgently required to understand the effects of pathogens on human alveoli. In recent years, advanced alveolar models have been developed^31,32,38–42^, and they employ 2D ALI and 3D organoid cultures because the physiological natural environment provides additional clinical relevance beyond the traditional 2D culture. However, the limited cell sources cannot support the reconstruction of tissue-level alveolar models. Existing alveolar cell lines, A549 and NCI-H441, are derived from human lung adenocarcinomas that fail to replicate normal alveolar functions such as epithelial barrier function and pulmonary surfactant production^43,44^. Although HPAEpiCs are an alternative source, the phenotype is rapidly lost during in vitro culturing because of the lack of standard culture protocols and culture media^9,10^. Several studies have used iPSC-derived alveolar cells^38,39^; however, the establishment of iPSC-derived cells is an extremely time-consuming process with the added challenge of genetic integrity^45,46^. Therefore, many questions remain unanswered regarding the in vitro culturing of human alveolar cells. For example, it is unclear whether (1) the cells support stable expansion, which can allow large-scale culturing for fundamental research; (2) the created alveolar models reproduce pathogen attack responses such as heterogeneous viral infections and tissue repair functions; and (3) the cells and created alveolar models have reasonable applications in translational medicine.

We developed a standardized HPAEpiC culture method that supports the long-term expansion and preservation for up to five passages (Figs. 1 and 2). The proposed SMC medium has great potential for the application of primary human alveolar epithelial cell culture owing to the possibility of application to any conventional cell culture method and overcoming yield limitations in large-scale HPAEpiC manufacturing. A stable cell source is critical for generating reproducible cell-based models for drug discovery and subsequent clinical trials. Thus, we not only developed a standardized culture medium for HPAEpiCs but also optimized the culture protocol for the ALI condition to determine the feasibility of large-scale alveolar modeling (Fig. 3). We demonstrated that the Sub condition promotes AT2-cell proliferation in the alveolar model, and we revealed that the alveolar models obtained under precultured Sub D7 and D14 conditions most closely resemble human alveoli. These findings provide important insights that will aid in the establishment of large-scale alveolar models.

We observed that the alveolar model reproduces the heterogeneous ACE2 and TMPRSS2 expression for the SARS-CoV-2 pseudovirus infection (Fig. 4c). Subsequently, we found that the virus infects both AT1 and AT2 cells (Fig. 4d, e), which is consistent with the histopathological pulmonary findings of COVID-19 autopsies^20,47–49^. Lentivirus-based pseudoviruses are useful for determining how the SARS-CoV-2 virus enters cells (Fig. 4g, h); however, a comprehensive investigation of the immune response or antiviral response requires the wild-type SARS-CoV-2 virus. We demonstrated that the proposed alveolar model has the potential to provide insight into the effects of the SARS-CoV-2 infection on alveolar epithelial function such as the activation of proinflammatory cytokines (IL-6 and IL-8) and the disruption of pulmonary surfactant (Fig. 4f and Supplementary Fig. 13). Although recent studies using induced pluripotent stem cell-derived AT2 cells have recorded similar data^38,39^, they may not be suitable for studying the pulmonary sequelae of COVID-19. Such AT2 cell-based alveolar models require 3D sphere cultures or co-cultures with fibroblasts^50^, which can generate confounding effects such as physical barriers of 3D hydrogel and non-endogenous fibroblast activation. Further, an alveolar model with AT2 cells limits the possibility of investigating the cellular complexity and regenerative function of human alveoli^51,52^. Therefore, our alveolar model provides an excellent opportunity to investigate the pulmonary sequelae of COVID-19, such as pulmonary fibrosis.

An important outcome of this study is the successful recapitulation of the plastic fates of AT1 and AT2 cells in alveolar homeostasis and post-injury repair. The activation and inhibition of Wnt signaling promotes wound healing rates (Fig. 5b–d), which is unexpected because Wnt-mediated niches are considered unidirectional routes for regulating the behavior of AT2 cell transdifferentiation and self-renewal^36^. The activation of Wnt signaling promotes AT2-cell maintenance and proliferation, whereas the inhibition of Wnt signaling inhibition promotes AT2-cell transdifferentiation into AT1 cells^37,53^. We found no differences in the numbers of AT1 and AT2 cells during the implementation of the Wnt-mediated repair model; however, we unexpectedly revealed that AT2 cells were concentrated at the healing boundary (Fig. 5e). This emphasizes the difference in the functional responses of AT2 cell-based and HPAEpiC-based alveolar models. A recent study on comprehensive single-cell mapping found that AT1 cells are essential signaling nodes during alveolar development and remodeling^15,16^, which includes the regulation of Wnt signaling. Thus, independent response experiments using only AT2 cells may not provide a comprehensive understanding of the complexity and plasticity of human alveoli^54^. In the TGF-β1-induced alveolar model, our study provided further evidence that the inhibition of Wnt signaling reduced the appearance of fibroblast-like cells and α-SMA^+^ cells while maintaining the phenotype of alveolar epithelial cells (Fig. 5f–h). This finding is consistent with a previous animal experiment^55^; however, our study provides additional insight that the preventive effect of pulmonary fibrosis via Wnt signaling can be attributed to the maintenance of the alveolar epithelial cell phenotype.

Thus far, there has been a consensus that both AT1 and AT2 cells contribute to human alveolus regeneration and plasticity^11–16^. In 2012, a classical follow-up study of a pneumonectomy patient showed a 64% increase in alveolar numbers over 15 years, thereby implying the high plasticity of human alveolar cells^56^. In general, AT2 cells are considered to serve as progenitor cells in alveolar regeneration, whereas AT1 cells are terminal cells. Further, recent studies identified Axin2^+^ AT2 and Il1r1^+^AT2 cells as major active contributors involved in the repair process after alveolar injury^36,53,57^. However, several studies showed that Hopx^+^ insulin-like growth factor binding protein (Igfbp)2^–^ AT1 cells also act as progenitor cells that can proliferate and differentiate into AT2 cells^14^. Importantly, AT1 cells have recently been identified as a distinct signaling hub^16^, which constitute an important source of ligands for establishing alveolar tissue homeostasis. Despite the extensive studies on alveolar epithelial cells^58,59^, mechanisms of alveolar epithelial injury and repair response remain poorly understood.

**Fig. 6:**
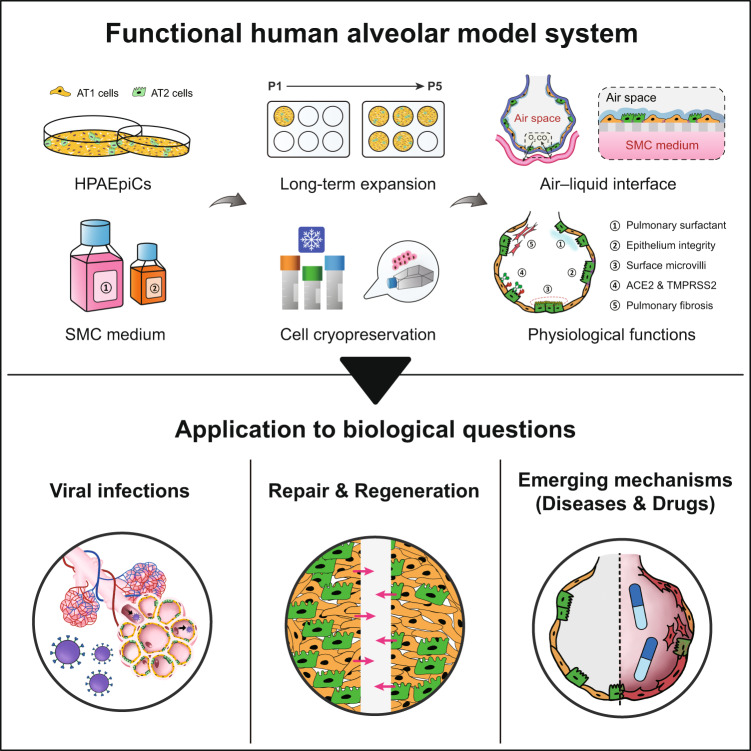
Schematic summary of functional human alveolar model and potential applications. In this study, a customized SMC medium was developed to support long-term HPAEpiC expansion and culture while maintaining the AT1 and AT2 cell phenotypes. During ALI modeling, the human alveolar model reproduced the key structural and functional characteristics, which include (1) pulmonary surfactant production, (2) epithelium integrity, (3) surface microvilli, (4) tissue-specific protein expression (ACE2, TMPRSS2), and (5) cellular regeneration and disease development. Following SARS‐CoV‐2 infection, the pseudovirus enters both the AT1 and AT2 cells, destroys the pulmonary surfactant (SPA), and induces IL-8 secretion. In the process of repairing the damaged alveolar model, XAV939 greatly improved wound healing and reduced fibroblast activation; thus, the mediation of Wnt signaling is crucial for alveolar regeneration. These applications highlight the fact that a developed human alveolar model system can help researchers investigate additional physiological responses, which enhances human lung research that includes viral infection studies and drug testing.

In conclusion, we proposed a strategy for HPAEpiC culturing, which not only represents an important milestone in lung research, but also highlights the emergence of a revolutionary alveolar model for translational medicine (Fig. 6). An established alveolar model recapitulates the physiological tissue-level complexity of human alveoli, which includes the AT1- and AT2-cell self-renewal and differentiation, pulmonary surfactant production, and alveolar epithelium integrity. Our alveolar model reproduces the clinical characteristics of human alveoli infected with SARS-CoV-2. We revealed that the regulation of Wnt signaling contributes to the alveolar repair function and effectively prevents the pathogenesis of pulmonary fibrosis after alveolar injury. Thus, this newly developed HPAEpiC culture method provides an unparalleled opportunity to investigate human alveolar plasticity, lung disease, and preclinical drug development.

## Methods

### HPAEpiC source and maintenance

HPAEpiCs were purchased from ScienCell Research Laboratories (ScienCell, 3200) and maintained using a modified protocol. In brief, HPAEpiCs isolated from eight different donors were used in the experiment, which include cells from four males, one female, and three unknowns (Supplementary Table 4). As a modified protocol, the HPAEpiCs were seeded on culture vessels coated with growth factor reduced (GFR) Matrigel (8.7 μg/cm^2^, Corning, 354230) at a density of 1 × 10^4^–1.5 × 10^4^ cells/cm^2^ and maintained in an alveolar epithelial cell medium (AEpiCM, ScienCell, 3201) supplemented with or without Jagged-1 peptide (JAG-1, AnaSpec, AS-61298), recombinant human Noggin protein (hNoggin, R&D Systems, 6057-NG), recombinant hFGF-10 protein (hFGF-10, R&D Systems, 345-FG), recombinant human keratinocyte growth factor (KGF)/FGF-7 protein (hFGF-7, R&D Systems, 251-KG), SB431542 (Sigma-Aldrich, 616464), CHIR99021 (Sigma-Aldrich, SML1046), and Y-27632 (Sigma-Aldrich, 688001). The culture medium was exchanged every 2–3 days, and cells were grown in a cell culture incubator (Thermo Scientific, 370) at 37 °C with 5% CO_2_.

### HPAEpiC passaging and cryopreservation

The cells were harvested using a 1:1 dilution of trypsin/ethylenediaminetetraacetic acid (EDTA; ScienCell, 0103) after reaching ~80–90% confluence. The cells were dissociated in two steps using trypsin/EDTA for the removal of a small number of spindle-shaped cells (mesenchymal-like cells) that appeared during the maintenance period. In brief, the cells were rinsed twice with Dulbecco’s phosphate-buffered saline (DPBS) following the aspiration of the culture medium. For the first dissociation, the cells were incubated with trypsin/EDTA at 25 °C for 2–3 min; this duration depended on the separation time of the spindle-shaped cells as observed in microscope images. For the second dissociation, the supernatant of the first dissociation was removed and the cells were incubated with fresh trypsin/EDTA at 37 °C for 6–8 min to detach the remaining cells from adherent surfaces. The cells were then incubated with an equal volume of trypsin neutralization solution (ScienCell, 0113) and centrifuged at 1200 rpm for 5 min to pellet the cells. Finally, the supernatant was removed and the resuspended cells were seeded on GFR Matrigel-coated T25 flasks or multi-well plates, as described above. For the cryopreservation of HPAEpiCs, the cells were resuspended in cryogenic storage vials using a SMC medium containing 7% dimethyl sulfoxide; they were then placed in liquid nitrogen following slow overnight freezing at –80 °C.

### In vitro model of primary human alveolar epithelium

A long-term lung alveolar model was cultured on Falcon^®^ 24-well Transwell inserts (Corning, 353095) to mimic the physiological environment of human alveoli under the ALI condition. The cells were seeded on GFR Matrigel-coated Transwell inserts at a high density of 5 × 10^4^–6 × 10^4^ cells/cm^2^ and maintained with the SMC medium in both the apical and basal chambers of the Transwell insert to create the model. The cells grew as a monolayer within the first 3 days. Culturing under submerged conditions in the SMC medium was continued for 7, 14, and 21 days to study the stability of the HPAEpiCs. These specimens were labeled Sub D7, Sub D14, and Sub D21, respectively. Following cultivation under submerged conditions, the ALI condition was initiated by aspirating the medium from the apical chamber and continuing to maintain the cells for 7 or 14 days for enhancing HPAEpiC maturation and polarization. These specimens were labeled ALI D7 and ALI D14, respectively. The culture medium was exchanged every 2–3 days, and the cells were grown in a cell culture incubator at 37 °C with 5% CO_2_.

### Infection of lung alveolar model with SARS-CoV-2 spike protein and pseudovirus

Prior to the infection of the lung alveolar cell model, the culture medium was replaced with a control medium. In total, 200-nM SARS-CoV-2 spike protein (ACROBiosystems, SPN-C52H4) was added to the apical chamber of the Transwell insert for 3 h to confirm the cell binding ability with the SARS-CoV-2 spike protein. Subsequently, the cells were collected for western blotting, with the signals being detected by an Amersham™ Imager 600 system (GE Healthcare). For viral infection experiments, the SARS-CoV-2 spike-pseudotyped lentivirus (enhanced green fluorescent protein (eGFP) or luciferase reporter) was purchased from the National RNAi Core Facility of Academia Sinica, Taiwan; its RNA sequence was determined. The pseudoviruses were added to the apical chamber of the Transwell insert at the indicated MOI for 1 day. The pseudoviruses were subsequently removed and the cells were cultured in the control medium under the ALI condition. Three days after infection, a GFP signal was observed in the infected alveolar model. The cells were then stained with specific antibodies and the proinflammatory cytokine response was analyzed. For the neutralization assay, the lung alveolar model was pretreated with 20-μg/ml anti-ACE2 antibodies (Abcam, ab87436) for 30 min before infection for blocking the ACE2 receptors on the cell surface.

### Treatment of lung alveolar model with lipopolysaccharide

Prior to treating the lung alveolar cell model (Sub D7 + ALI D7), the culture medium was replaced with the control medium. Then, 100 μg/ml LPS (Sigma-Aldrich, L4391) was added to the apical chamber of the Transwell insert for 24 h. The cells were stained with specific antibodies to observe the lung alveolar model barrier function.

### Wound healing assay

The lung alveolar model for the ALI condition (Sub D7 + ALI D7) established as described above was used in a study of scratch wound healing. The cells on the Transwell insert were gently scratched with a sterile 200-μl pipette tip (approximate outer orifice diameter: 800–900 μm) to create a straight wound region. The cells were then rinsed twice with DPBS to remove any detached cells and cultured under the ALI condition with the control medium containing 10-μM XAV939 (Sigma-Aldrich, X3004), CHIR99021, or SB431542, in the presence and absence of 5 ng/ml TGF-β1 (R&D Systems, 240-B-002). The wound healing process was photographed at 24 and 48 h post-scratching. Cells were then stained with specific antibodies to analyze the wound healing ability.

### Immunofluorescence staining

Following two washes with DPBS, cells were fixed and permeabilized with the Cytofix/Cytoperm Solution (BD Biosciences, 554722) for 15 min. The fixed cells were incubated with a blocking buffer composed of DPBS containing 1% bovine serum albumin and 5% fetal bovine serum for 30 min; then, they were incubated with primary antibodies (Supplementary Table 5) at room temperature for 2 h or overnight at 4 °C. This was followed by incubation with the corresponding secondary antibodies (Supplementary Table 6) for 2 h at room temperature. The cell nuclei were counterstained with Hoechst 33342 (Invitrogen, 62249) or 4′,6-diamidino-2-phenylindole, and the cells were mounted on microscope slides with an anti-fade reagent (VECTASHIELD, H-1000, and H-1200). Fluorescence imaging was performed using a confocal laser-scanning microscope (Leica, TCS SP8) and an automated high-content imaging system (Molecular Devices, ImageXpress Micro 4). Images were analyzed using MetaXpress^®^ software (Molecular Devices, version 6.5.3.427).

### Gene expression analysis

The total RNA was extracted using a RNeasy mini kit (Qiagen, 74104) and RNAse-Free DNase set kit (Qiagen, 79254); it was then reverse transcribed into cDNA using a high-capacity cDNA reverse transcription kit (Applied BiosystemsTM, 4368814). The DNA and cDNA quality and quantity were assessed using a NanoPhotometer^®^ (Implen, NP80). Quantitative real-time polymerase chain reactions (PCRs) were performed using a StepOne^TM^ real-time PCR system (Applied Biosystems^TM^) and PowerUP^TM^ SYBR^TM^ green master mix (Applied Biosystems^TM^, A25918). Glyceraldehyde 3-phosphate dehydrogenase was used as internal control, and the relative gene expression was calculated using the 2^−ΔΔCT^ method. Primers were designed using the GenScript^®^ real-time PCR primer design software or NCBI-BLAST software; the sequences are listed in Supplementary Table 7.

### Scanning electron microscopy

The transparent porous membrane supporting cell culture was excised from the Transwell inserts with a scalpel; the cells were fixed with 2.5% glutaraldehyde for 1 h. Then, the cells were washed three times with DPBS and sequentially dehydrated in a graded ethanol series (35, 50, 70, 85, 95, and 98%) for 15 min each. Finally, the cells were immersed in hexamethyldisilazane for 5 min. They were then air dried in an extraction cabinet at room temperature overnight. The samples were sputter-coated with gold at 30 mA for 60 s and imaged using a high-resolution field emission scanning electron microscope (JEOL, JSM-7610F) at 5 kV accelerating voltage to observe cell morphology.

### Phosphatidylcholine analysis

The PC concentrations of lung alveolar cell models were examined using the PC assay kit (Abcam, ab83377). In brief, the cells were washed with DPBS, resuspended in the assay buffer, and homogenized by pipetting up and down. Samples were then centrifuged at 12,000 rpm for 5 min at 4 °C, and the collected supernatant was mixed with an assay buffer, PC hydrolysis enzyme, PC development mix, and OxiRe probe. The PC concentration was determined by measuring the absorbance at 570 nm using a microplate reader (ChroMate-4300).

### Western blotting

Cell lysates were harvested in a 1X radioimmunoprecipitation assay buffer (Abcam, ab156034) containing an EDTA-free protease inhibitor cocktail (Sigma-Aldrich, 40693159001), which was quantified using the Pierce^TM^ bicinchoninic acid protein assay kit (Thermo Scientific, 23225) and mixed with a 4X loading dye. Samples were then separated using sodium dodecyl sulfate–polyacrylamide gel electrophoresis and transferred to a polyvinylidene fluoride membrane. The membranes were blocked with 5% (w/v) nonfat dried milk in TBST (20 mM Tris base, pH 7.4, 150 mM NaCl, 0.1% (v/v) Tween-20) for 1 h and incubated with the primary antibodies (Supplementary Table 8) overnight at 4 °C. Incubation with the corresponding secondary antibodies (Supplementary Table 9) was then performed for 1 h. The immunoreactive bands from each sample were visualized using immobilon western chemiluminescent horseradish peroxidase (HRP) substrate (Merck Millipore, WBKLS0500) and the Amersham™ imager 600 system (GE Healthcare).

### Inflammation cytokines analysis

Control medium (100 μl) was added to the apical chamber of the Transwell insert for 15 min and collected for cytokine analysis. IL-8 and IL-6 concentrations were examined using the human IL-8 uncoated enzyme-linked immunosorbent assay (ELISA) kit (Invitrogen, 88-8086) and the human IL-6 DuoSet ELISA kit (R&D Systems, DY206), respectively. In brief, 96-well ELISA plates were coated with specific capture antibodies at 4 °C overnight and incubated with blocking buffer at the room temperature for 1 h. Standard solutions and collected samples were added to the appropriate wells, and ELISA plates were then incubated sequentially with the detection antibody and Avidin-HRP. Analyte concentrations were determined by adding tetramethylbenzidine solution and measuring the mixture absorbance at 450 nm using a microplate reader.

### Statistics and reproducibility

GraphPad prism (GraphPad software, version 8.0.2) was used to analyze the data and to plot the graphs. The data were expressed as mean ± the standard error of the mean values. The statistical differences were analyzed using the unpaired Student’s *t* test and one-way or two-way analysis of variance tests; the values were reported as significant if the *p* values were <0.05.

### Reporting summary

Further information on research design is available in the Nature Research Reporting Summary linked to this article.

## Supplementary information

Supplementary Information

Description of Additional Supplementary Files

Supplementary Data 1

Reporting Summary

## Data Availability

All data generated or analyzed during this study are included in this published article and Supplementary Information, or are available from the corresponding author upon reasonable request. The uncropped and unedited blot images are available in Supplementary Figs. 15 and 16. The source data underlying the graphs and charts in the figure are provided in Supplementary Data 1.
