# Supplementary material for: Tissue-level alveolar epithelium model for recapitulating SARS-CoV-2 infection and cellular plasticity
Source: Commun Biol. 2022 Jan 19;5:70. doi: 10.1038/s42003-022-03026-3 (PMC8770515; doi:10.1038/s42003-022-03026-3)
Supplement: Supplementary file 1 — Supplementary Information [file 42003_2022_3026_MOESM1_ESM.pdf]

Supplementary Information

Tissue-level alveolar epithelium model for recapitulating SARS-CoV-2 infection and cellular plasticity

Jia-Wei Yang[1,2], Yu-Rou Lin[2], Ying-Ling Chu[2], Johnson H.Y. Chung[3], Huai-En Lu[4], Guan-Yu Chen[1,2,5,6*]

[1]Department of Electrical and Computer Engineering, College of Electrical and Computer Engineering, National Yang Ming Chiao Tung University, Hsinchu, Taiwan.

[2]Institute of Biomedical Engineering, College of Electrical and Computer Engineering, National Yang Ming Chiao Tung University, Hsinchu, Taiwan.

[3]ARC Centre of Excellence for Electromaterials Science, Intelligent Polymer Research Institute, University of Wollongong, Wollongong, NSW, Australia.

[4]Bioresource Collection and Research Center, Food Industry Research and Development Institute, Hsinchu, Taiwan.

[5]Department of Biological Science and Technology, National Yang Ming Chiao Tung University, Hsinchu, Taiwan.

[6]Institute of Biomedical Engineering, College of Electrical and Computer Engineering, National Chiao Tung University, Hsinchu, Taiwan.

*Corresponding author

Email: guanyu@nctu.edu.tw

Phone: (886) 3-573-1920

FAX: (886) 3-573-1672

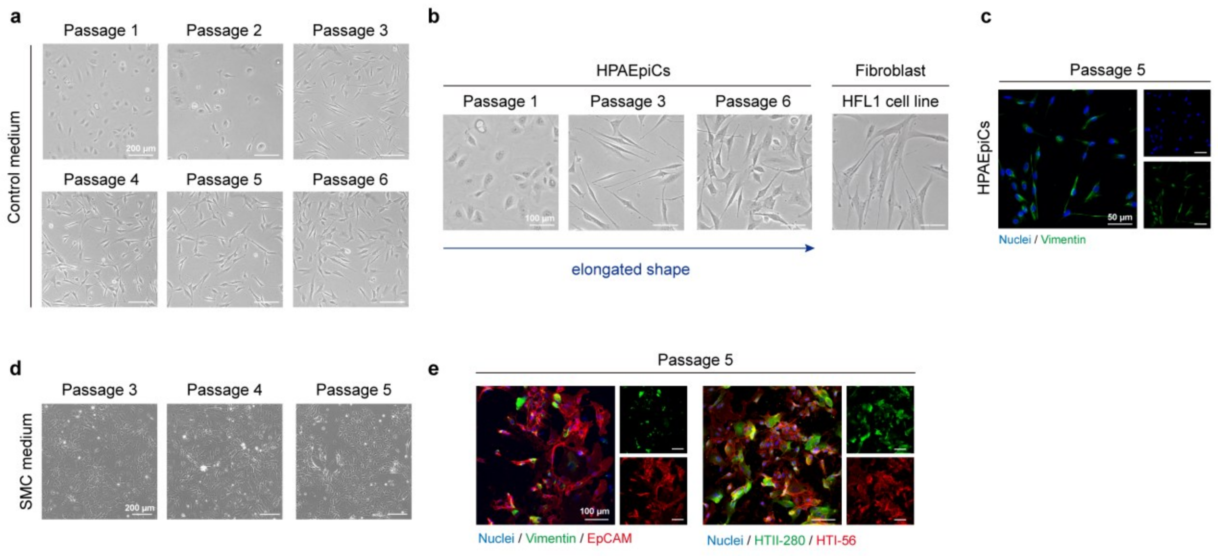

Supplementary Figure 1. HPAEpiCs cultured with control and SMC medium. (**a**) Bright-field microscope images showing HPAEpiC elongation with increasing passage number. **(b)** The elongated HPAEpiCs exhibited similar characteristics to the human lung fibroblast cell line, HLF-1. (**c**) The HPAEpiCs were stained with Vimentin (green) antibody at Passage 5, which indicated that almost all cells were Vimentin$^+$ cells. (**d**) Bright-field microscope images showing HPAEpiC morphology maintenance during 5 passages. (**e**) The HPAEpiCs were stained with Vimentin (green), EpCAM (red), HTII-280 (green), and HTI-56 (red) antibodies at Passage 5. From the images, the HPAEpiCs maintained the epithelial cell phenotype.

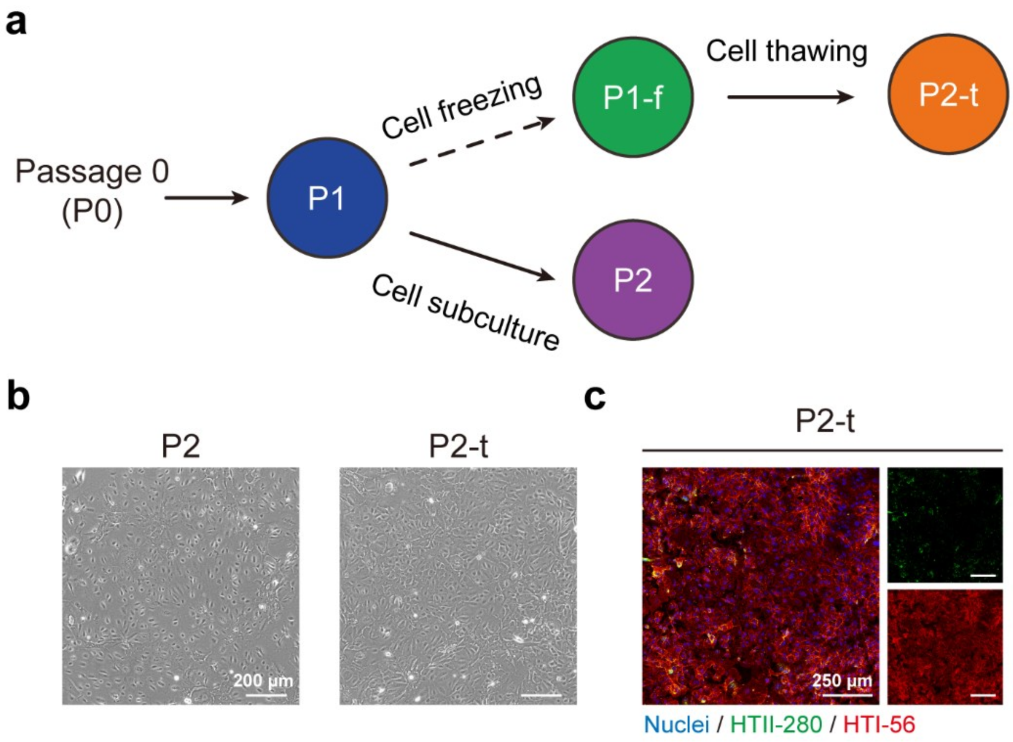

Supplementary Figure 2. SMC medium for HPAEpiC freezing and thawing. (**a**) Schematic diagram of experiment on cell passaging culture, freezing, and thawing. (**b**) Bright-field microscope images showing maintenance of epithelial cell morphology by frozen–thawed HPAEpiCs. (**c**) The frozen-thawed HPAEpiCs were stained with HTII-280 (green) and HTI-56 (red) antibodies, showing that the AT1 and AT2 cell phenotypes were maintained in the SMC medium.

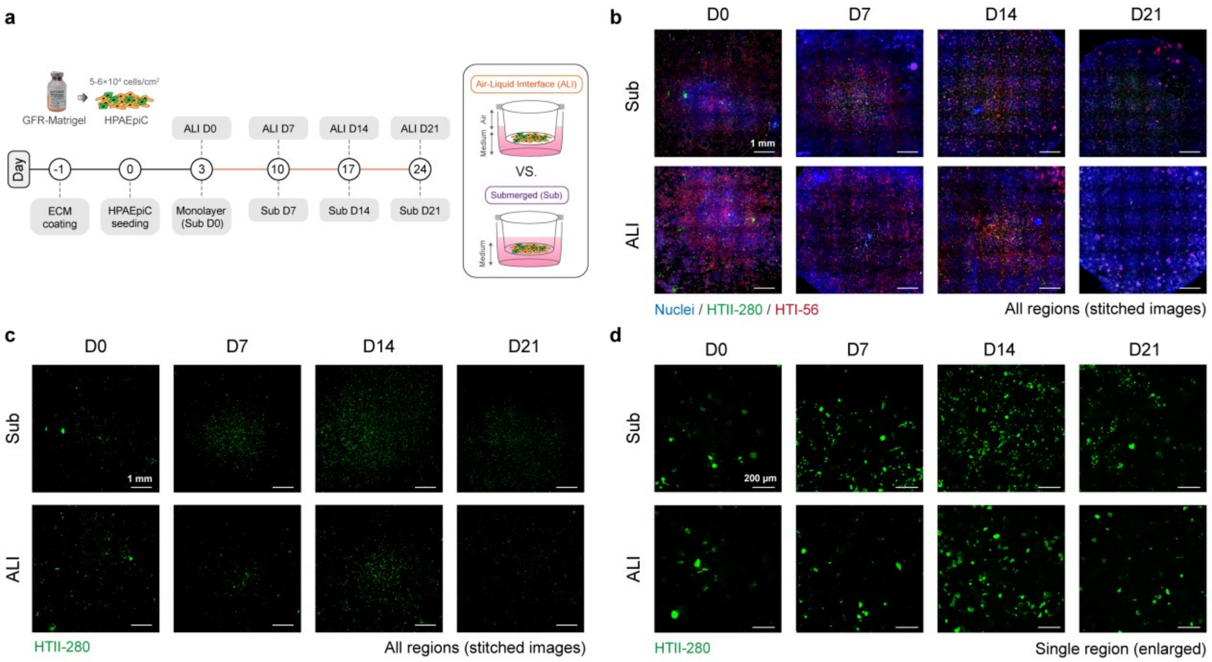

Supplementary Figure 3. Human alveolar models established under continuous Sub or ALI conditions. (**a**) Time-line flowchart showing HPAEpiC culture steps under continuous Sub or ALI conditions. (**b–d**) The HPAEpiCs were stained with HTII-280 (green) and HTI-56 (red) antibodies under Sub and ALI culture conditions at 0, 7, 14, and 21 days. The images are shown as whole regions of stacked images (b) and HTII-280$^+$ cell images (c), as well as local regions of HTII-280$^+$ cell images (d).

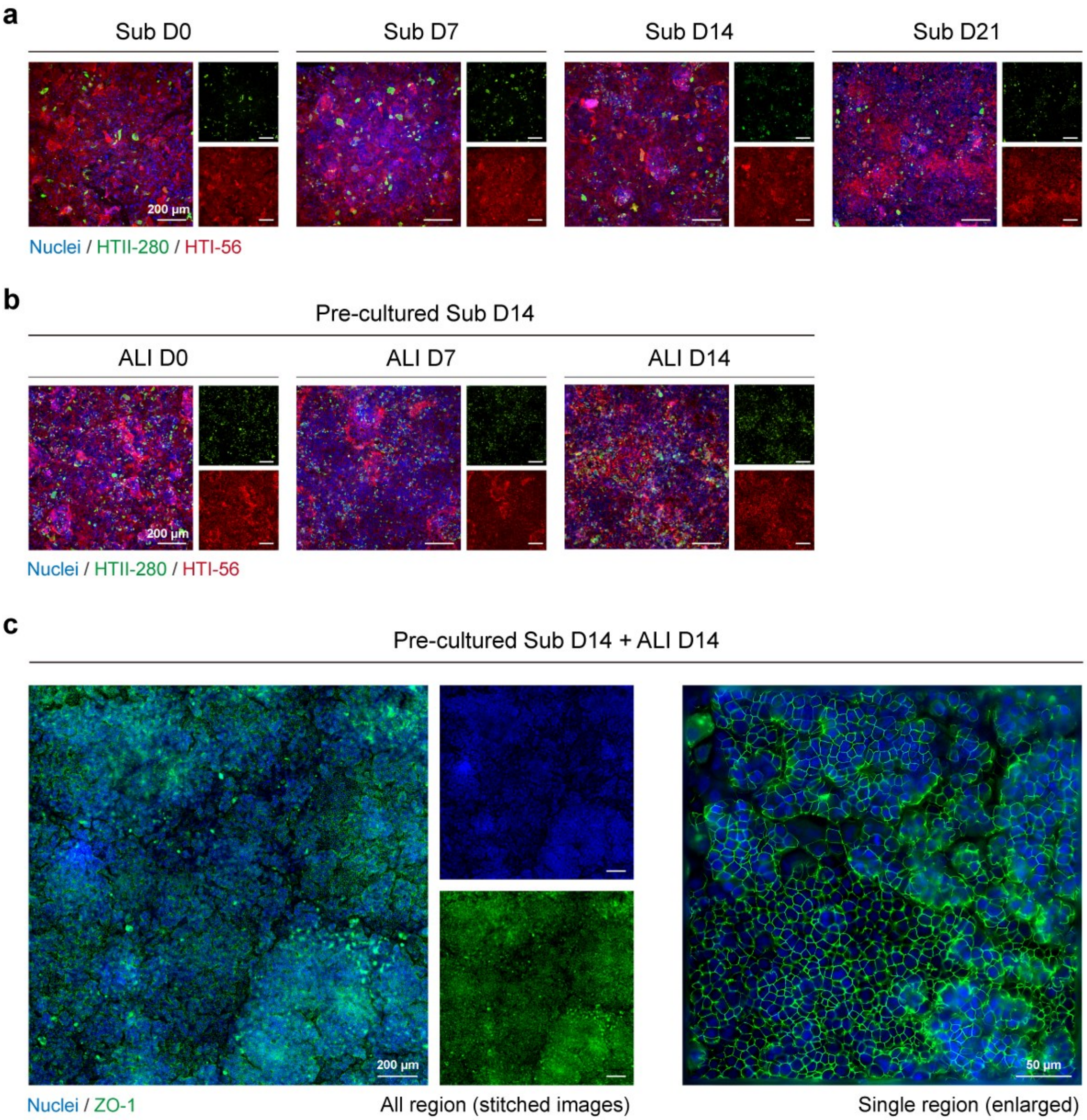

Supplementary Figure 4. Human alveolar models established under continuous Sub condition or two-staged Sub and ALI conditions. (**a**) The HPAEpiCs were stained with HTII-280 (green) and HTI-56 (red) antibodies under Sub conditions at 0, 7, 14, and 21 days. (**b**) The HPAEpiCs were stained with HTII-280 (green) and HTI-56 (red) antibodies under pre-cultured Sub D14+ALI D0, D7, D14 conditions. These images show that the human alveolar model forms a more compact monolayer under ALI conditions. (**c**) The HPAEpiCs were stained with ZO-1 antibodies under pre-cultured Sub D14+ALI D14 condition.

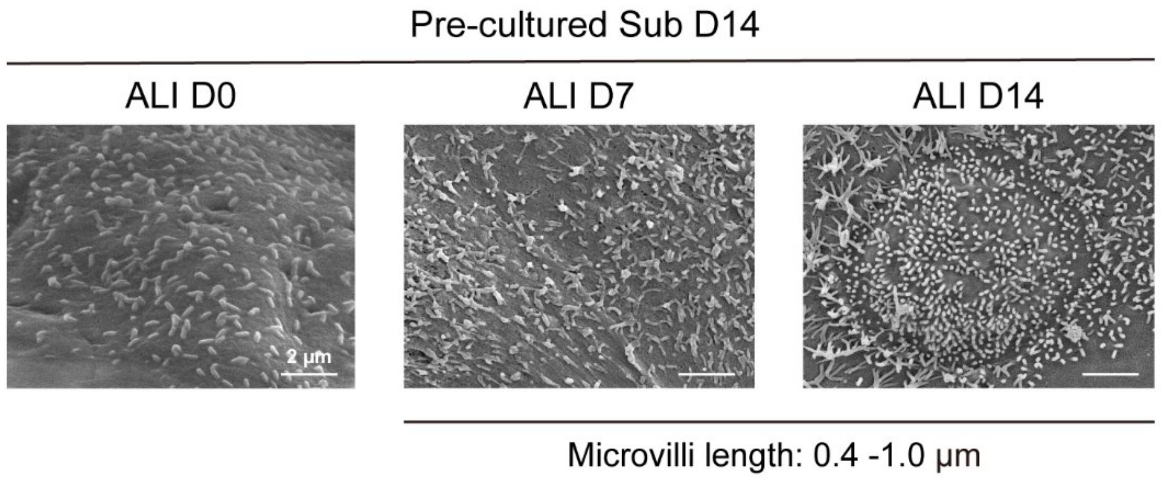

Supplementary Figure 5. Microvilli of human alveolar models established under ALI conditions. The HPAEpiCs were cultured under pre-cultured Sub D14+ALI D0, D7, D14 conditions. The scanning electron microscope images showed that the HPAEpiC microvilli increased under ALI conditions with lengths between 0.4 and 1.0 μm.

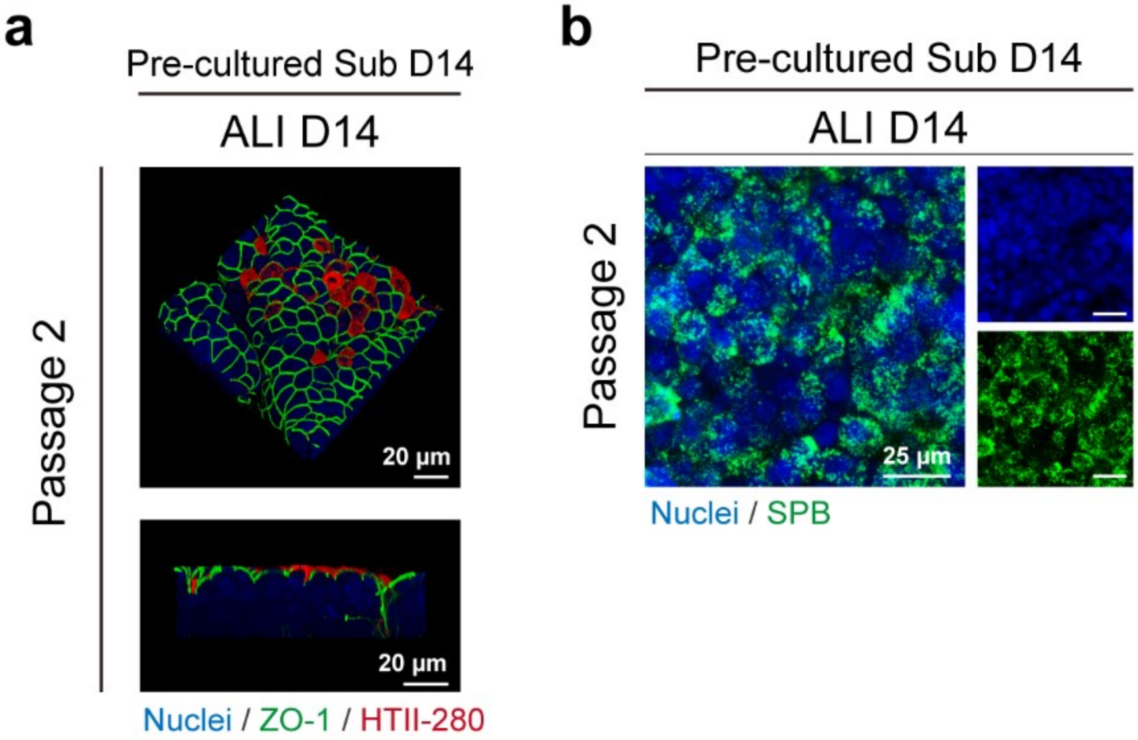

Supplementary Figure 6. Cell functions of human alveolar model established with passaged HPAEpiCs. (**a**) The HPAEpiCs were stained with ZO-1 (green) and HTII-280 (red) antibodies, showing that the alveolar model exhibited integrity barrier characteristics containing AT2 cells. (**b**) The HPAEpiCs were stained with SPB (green) antibody, showing that the alveolar model produced homogeneous surfactant-specific protein.

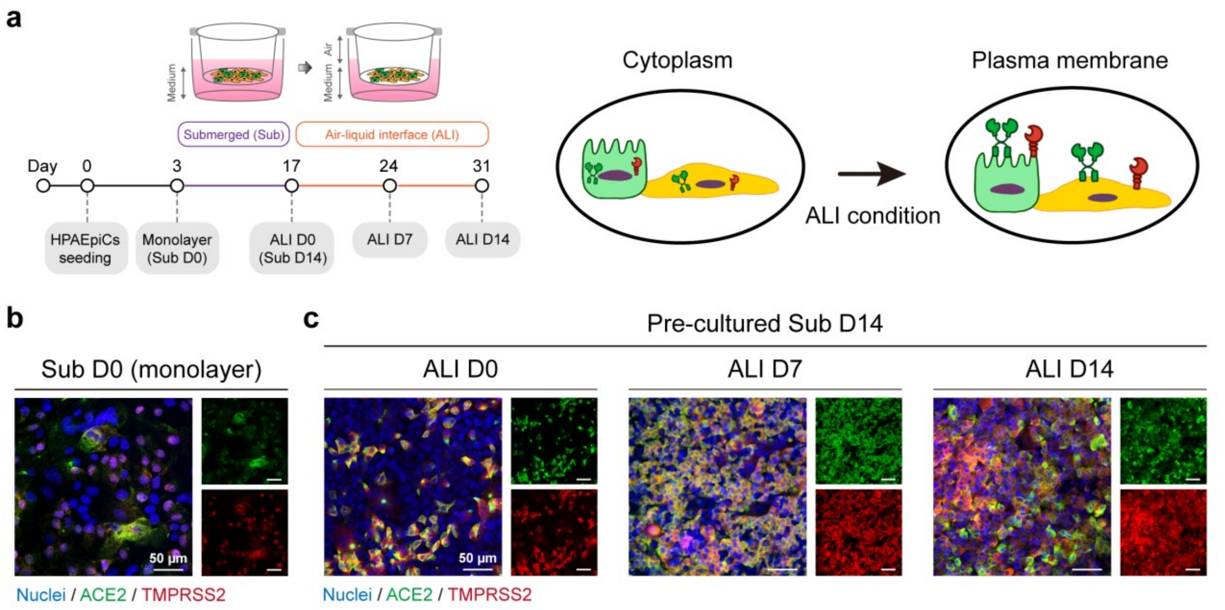

Supplementary Figure 7. ACE2 and TMPRSS2 expression during establishment of human alveolar models. (**a**) Schematic diagram showing abundant expression of ACE2 and TMPRSS2 in human alveolar model under ALI conditions. (**b**) The HPAEpiCs were stained with ACE2 (green) and TMPRSS2 (red) antibodies under the Sub D0 condition. From the images, the ACE2 and TMPRSS2 expression was mostly located in the cell nuclei. (**c**) After the ALI condition, the ACE2 and TMPRSS2 expression increased substantially and had a certain co-localization in the human alveolar model.

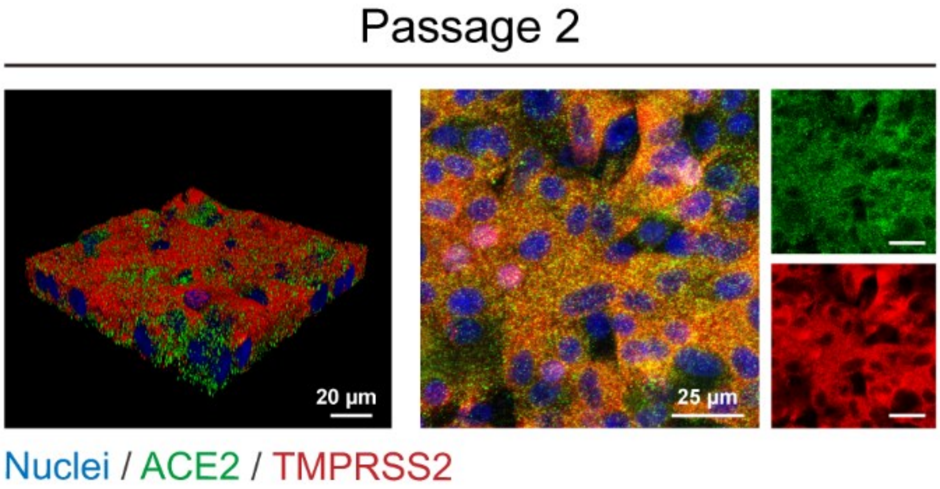

Supplementary Figure 8. ACE2 and TMPRSS2 expression in human alveolar models established with passaged HPAEpiC cells. The HPAEpiCs were stained with ACE2 (green) and TMPRSS2 (red) antibodies, showing the feasibility of establishing a large-scale alveolar model expressing ACE2 and TMPRSS2.

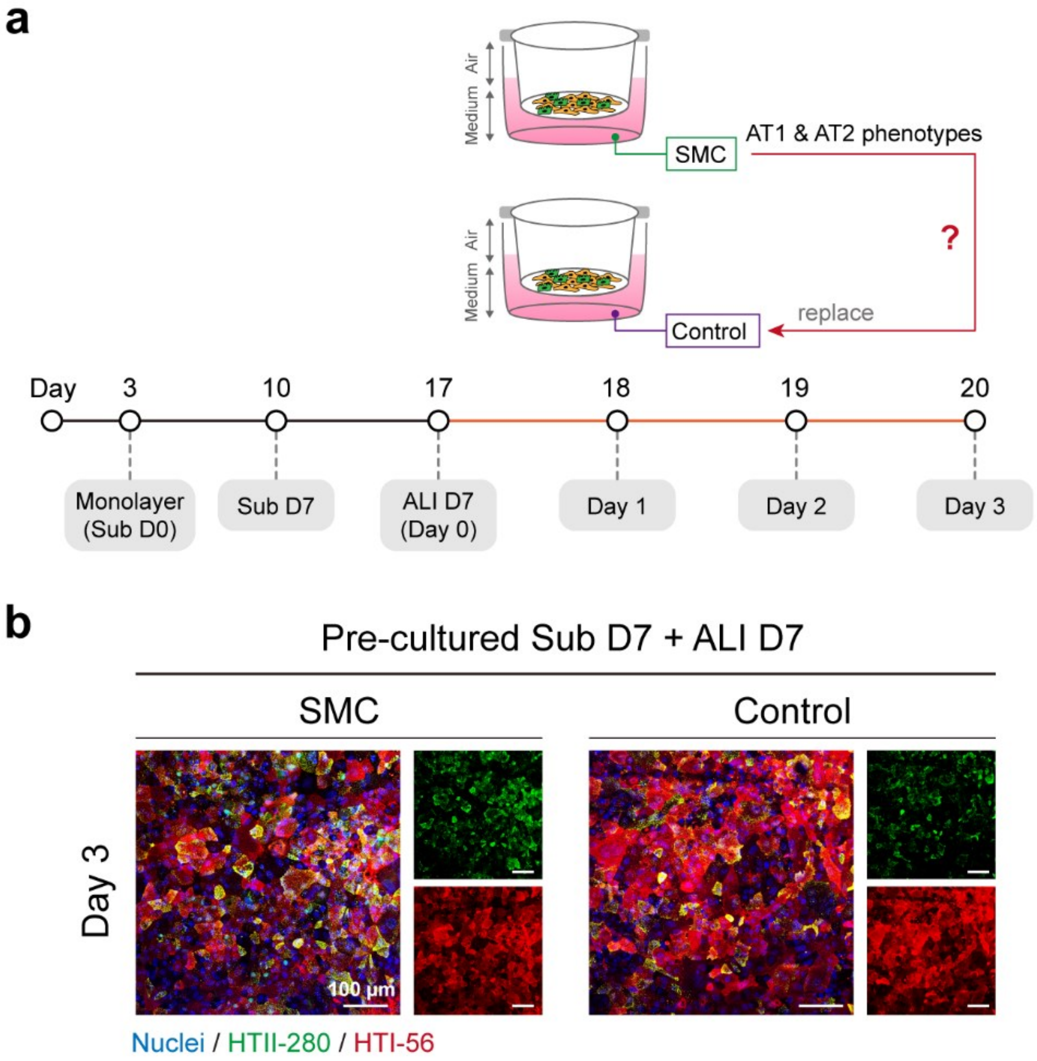

Supplementary Figure 9. Replacement of SMC culture medium with control after establishment of human alveolar model. (**a**) Time-line flowchart showing HPAEpiC culture steps with control or SMC medium after establishment of human alveolar model. (**b**) The HPAEpiCs were stained with HTII-280 (green) and HTI-56 (red) antibodies after 3 days of medium replacement. From the images, the human alveolar model maintained the specific protein expression of the AT1 and AT2 cells even when the SMC medium was replaced with the control medium.

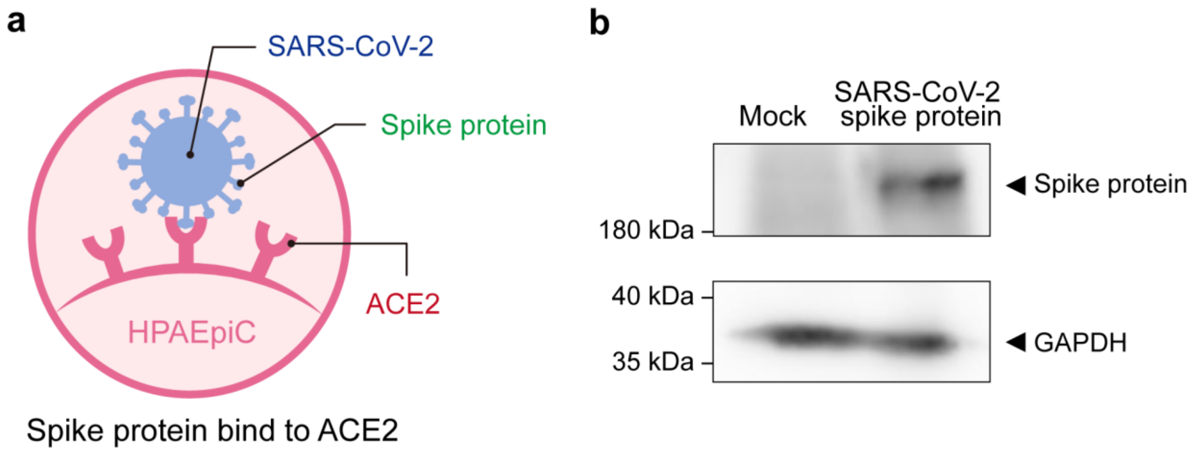

Supplementary Figure 10. Interaction between SARS-CoV-2 spike protein and human alveolar model. (**a**) Schematic diagram of interaction between SARS-CoV-2 spike protein and ACE2 receptor. (**b**) After 6 h treatment, the western blotting data showed a binding interaction between the SARS-CoV-2 spike protein and human alveolar model.

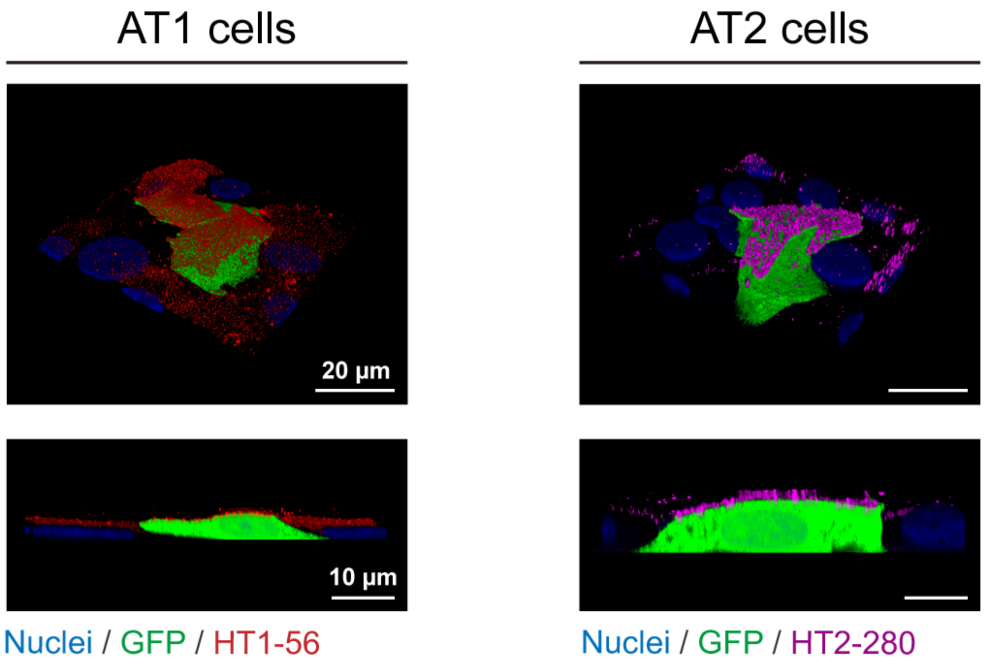

Supplementary Figure 11. SARS-CoV-2 pseudovirus infection in human alveolar model. After 3 days of SARS-CoV-2 pseudovirus infection (GFP reporter), the HPAEpiCs were stained with HTI-56 (red) or HTII-280 (purple) antibodies. The 3D confocal images reveal that both the AT1 (HTI-56^+) and AT2 cells (HTII-280^+) were infected with the SARS-CoV-2 pseudovirus.

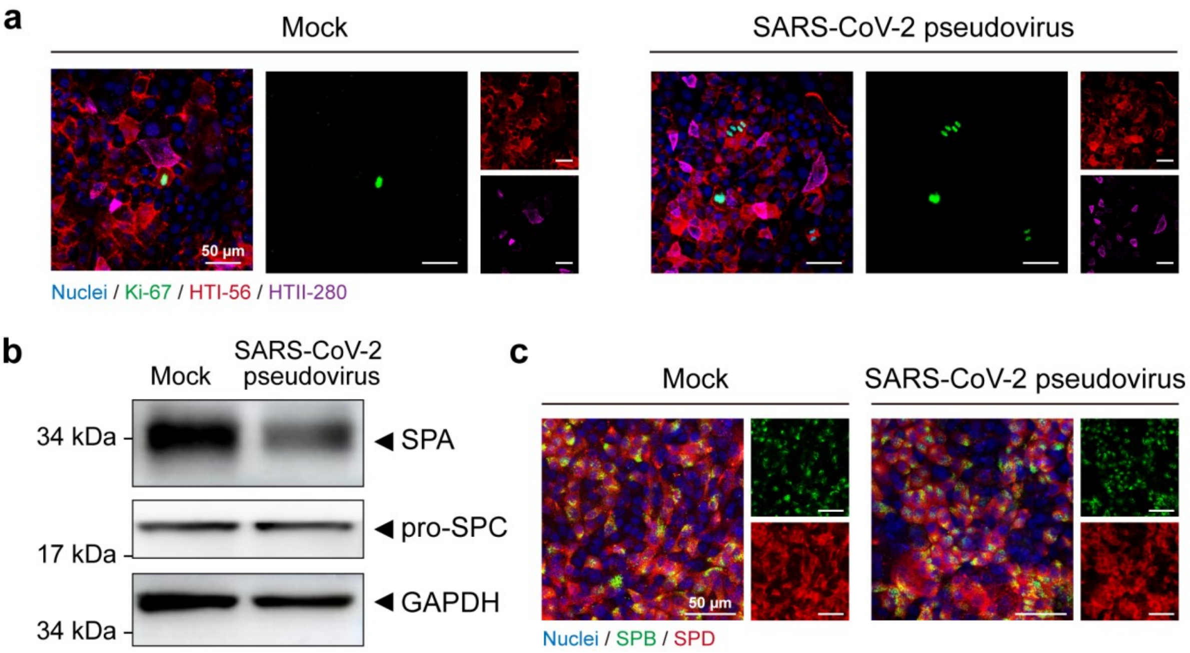

Supplementary Figure 12. Cell function of human alveolar model after SARS-CoV-2 pesudovirus infection. (**a**) After 3 days of infection, the cell lysates were collected for western blot analysis to detect the HPAEpiC SPA and pro-SPC proteins. The SPA expression decreased obviously after SARS-CoV-2 pseudovirus infection. (**b**) The HPAEpiCs were stained with SPB (green) and SPD (red) antibodies; there were no obvious differences in SPB and SPD expression after pseudovirus infection. (**c**) The HPAEpiCs were stained with Ki-67 (green), HTI-56 (red), and HTII-280 (purple) antibodies. From the images, there were more proliferating cells (Ki-67^{+}) in the pseudovirus-infected alveolar model.

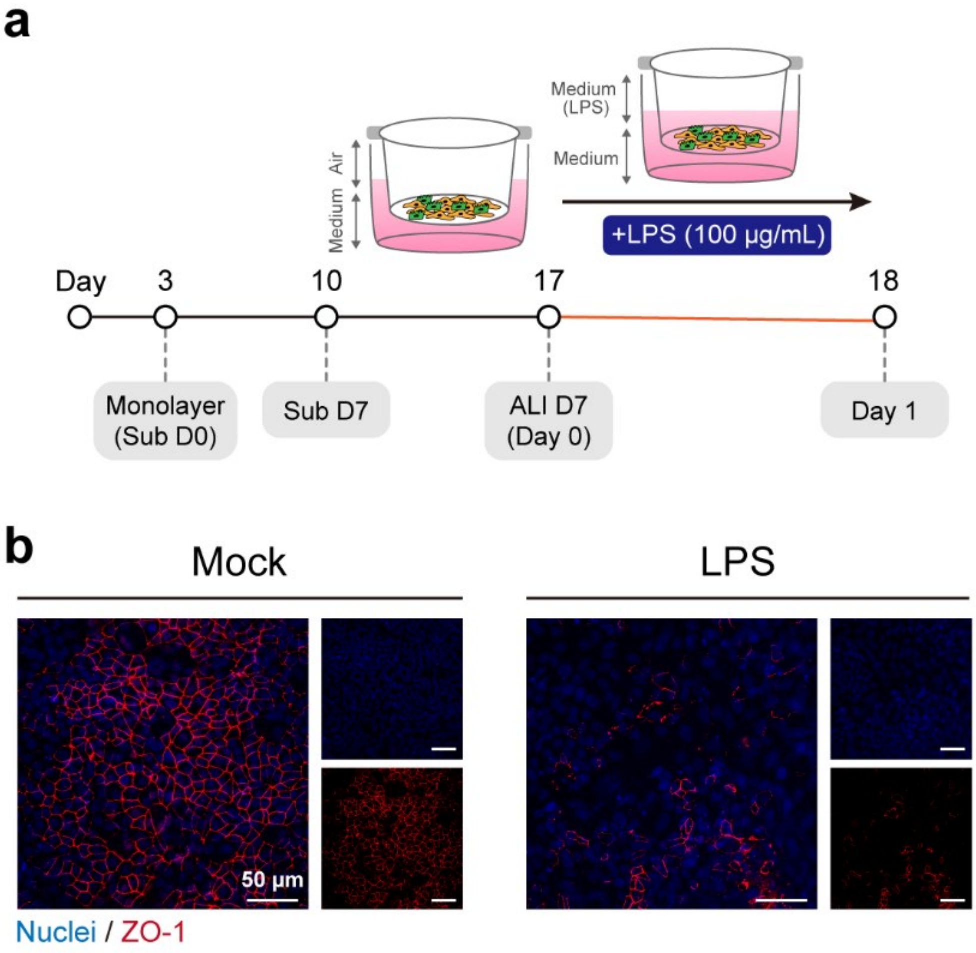

Supplementary Figure 13. Human alveolar model treated with lipopolysaccharide (LPS). (a) Time-line flowchart showing HPAEpiC culture steps during LPS treatment. (**b**) After LPS treatment, the HPAEpiCs were stained with ZO-1 (red) antibody. From the images, the ZO-1 signal was almost absent after LPS treatment, suggesting that the barrier function of the human alveolar model was impaired.

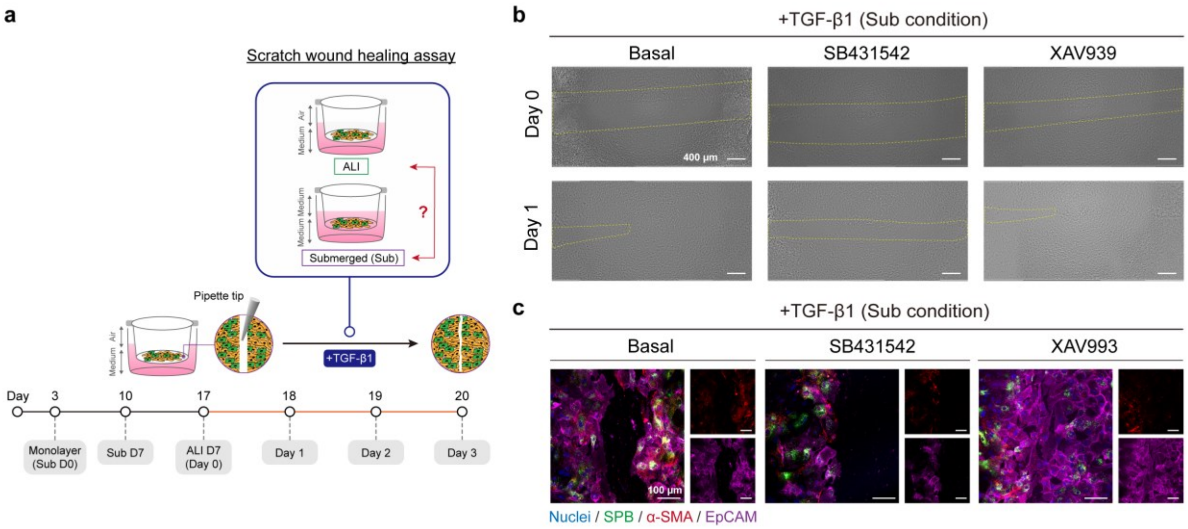

Supplementary Figure 14. Repair response of damaged human alveolar models under Sub condition. (**a**) Time-line flowchart showing HPAEpiC culture steps during wound healing. (**b**) The human alveolar models were co-treated with TGF-β1 and SB431542 or XAV939 for 1 day. From the images, HPAEpiC treatment with XAV939 enhanced wound healing in the alveolar model, with almost complete healing being achieved within a day. (**c**) The HPAEpiCs were stained with SPB (green), α-SMA (red), and EpCAM (purple) antibodies. From the images, XAV939 maintained the highest EpCAM expression during wound healing in the human alveolar model.

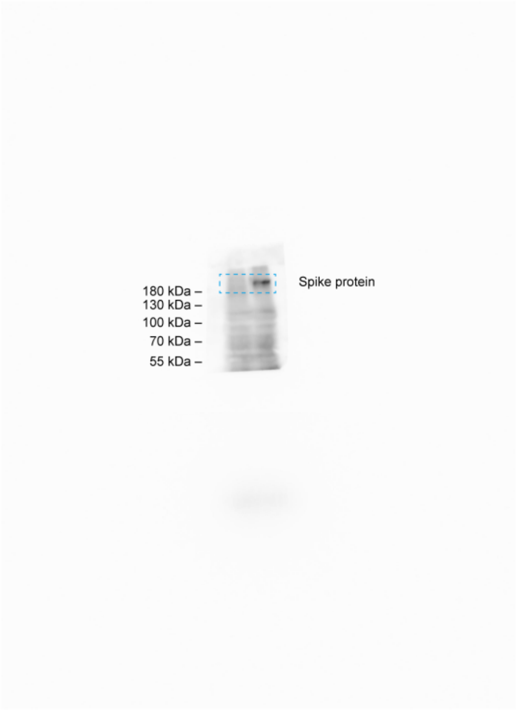

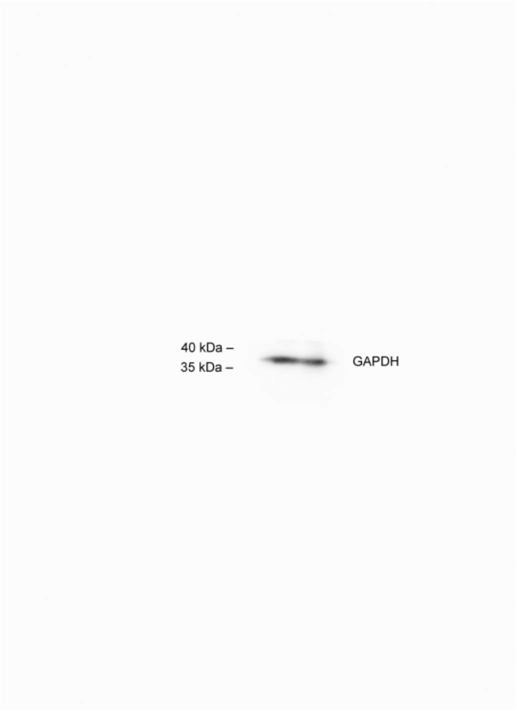

Supplementary Figure 15. Uncropped and unedited images for Supplementary Figure 10b.

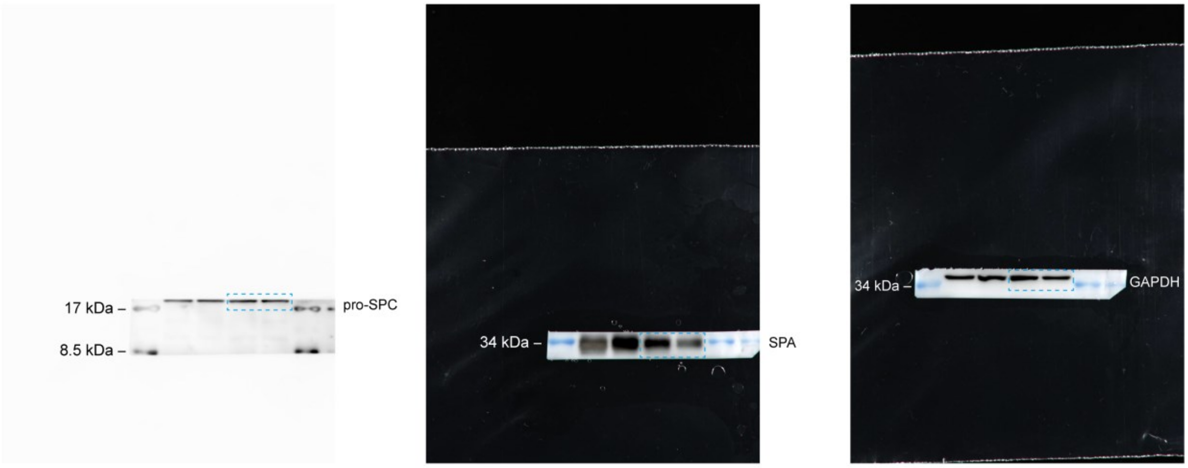

Supplementary Figure 16. Uncropped and unedited images for Supplementary Figure 12b.

Supplementary Table 1. The regulatory functions of medium additives on HPAEpiC growth behavior

Medium additives	Description	Signaling pathways	Primary functions	Reference
JAG-1	This peptide is a fragment of the JAG-1 protein, which is a Notch ligand.	Notch signaling ↑	Alveolar cell maintenance	1
hNoggin	This recombinant protein is an antagonist of bone morphogenetic proteins (BMPs).	BMP signaling ↓	AT2 cells self-renewal	2
SB431542	This small molecule is a selective inhibitor of transforming growth factor-β type I receptor (TGF-β RI).	TGF-β signaling ↓	Prevention of AT2 cells transdifferentiation into mesenchymal cells	3
hFGF-10	This recombinant protein is a subgroup of fibroblast growth factor (FGF).	FGF signaling ↑	AT2 cells maintenance	4
hFGF-7	This recombinant protein is a member of the FGF family, also known as keratinocyte growth factor (KGF).	FGF signaling ↑	AT1 and AT2 cells maintenance;<br>AT2 cells proliferation	5, 6
CHIR99021	This small molecule is an aminopyrimidine derivative that acts as an inhibitor of glycogen synthase kinase 3 (GSK-3).	Wnt signaling ↑	AT2 cells maintenance;<br>Inhibition of AT2 to AT1 cells transdifferentiation	7, 8
Y-27632	This small molecule is a selective inhibitor of Rho-associated coil kinase (ROCK).	RhoA/ROCK signaling ↓	Cells avoid anoikis	9

Supplementary Table 2. The components and parameters of HPAEpiC cell culture medium

Medium additives	Final concentration	Control	SMC	– JAG-1	– hNoggin	– SB431542	– hFGF-10	– hFGF-7	– CHIR99021
AEpiCM	1 X	+	+	+	+	+	+	+	+
JAG-1	1 µM	-	+	-	+	+	+	+	+
hNoggin	100 ng/ml	-	+	+	-	+	+	+	+
SB431542	10 µM	-	+	+	+	-	+	+	+
hFGH-10	100 ng/ml	-	+	+	+	+	-	+	+
hFGH-7	100 ng/ml	-	+	+	+	+	+	-	+
CHIR99021	3 µM	-	+	+	+	+	+	+	-
Y-27632 #	10 µM	-	+	+	+	+	+	+	+

Supplementary Table 3. The comparison of our alveolar model with human-related biological properties

	Human alveolar epithelial tissue	Our in vitro alveolar model	A549 cell line
**Cell-type composition**	AT1 and AT2 cells	AT1 and AT2 cells	AT2 cells
**AT2 cells proportion**	~ 15%	~ 10%	100%
**Surfactant proteins production**	SPA, SPB, SPC and SPD	SPA, SPB, pro-SPC and SPD	SPB, SPC and SPD
**Epithelial barrier integrity**	Well	Well	Weak
**Microvilli length**	~ 0.5 - 1.0 μm	~ 0.4 - 1.0 μm	None
**SARS-CoV-2 infection**	AT1 and AT2 cells	AT1 and AT2 cells	Non-permissive
**Reference**	[10-14]	In this study	[15-20]

Supplementary Table 4. The information of the HPAEpiCs used in the experiment

Lot. No.	Sex	Age (gestation)
28272	Unknown	20 weeks
28696	Male	20 weeks
29464	Male	19 weeks
29657	Female	21 weeks
30105	Male	20 weeks
30869	Male	20 weeks
31476	Unknown	20 weeks
31486	Unknown	20 weeks

Supplementary Table 5. The primary antibodies used for immunostaining

Primary anti-bodies	Company	Catalog number	Dilution
α-SMA	Cell Signaling Technology	36110S	1:100
ACE2	Abcam	ab87436	1:100
AQP5	Santa Cruz Biotechnology	sc-514022 AF594	1:100
CK18	BioLegend	628408	1:100
EpCAM	Cell Signaling Technology	5447S	1:400
EpCAM	BioLegend	324214	1:100
HTI-56	Terrace Biotech	HT1-56	1:50
HTII-280	Terrace Biotech	HT2-280	1:50
PDPN	Abcam	ab10288	1:100
pro-SPC	Merck Millipore	AB3786	1:200
SOX9	Abcam	ab196184	1:100
SPA	Abcam	ab51891	1:200
SPB	Thermo Scientific	MA1-204	1:100
SPD	Bioss	BS-1583R	1:100
TMPRSS2	Santa Cruz Biotechnology	sc-515727	1:100
Vimentin	Cell Signaling Technology	9854S	1:400
ZO-1	Thermo Scientific	339188	1:200
ZO-1	Cell Signaling Technology	13663S	1:400

Supplementary Table 6. The secondary antibodies used for immunostaining

Secondary antibodies	Company	Catalog number	Dilution
Alexa Fluor® 488/IgG	Jackson ImmunoResearch	115-545-062	1:200
Alexa Fluor® 488/IgG	Jackson ImmunoResearch	111-545-003	1:200
Alexa Fluor® 488/IgM	Thermo Scientific	A-21042	1:200
Alexa Fluor® 555/IgG1	Thermo Scientific	A-21127	1:200
Alexa Fluor® 633/IgM	Thermo Scientific	A-21046	1:200
Alexa Fluor® 647/IgG	Jackson ImmunoResearch	115-605-003	1:200
Cy3/IgG	Merck Millipore	AP132C	1:200

Supplementary Table 7. The primers sequences for qRT-PCR

Genes	Forward (5'→3')	Reverse (5'→3')
Vimentin	GGATGTTTCCAAGCCTGACC	TTCTTGGCAGCCACACTTTC
EpCAM	GGCTCTTTAAGGCCAAGCAG	CCAGTAGGTTCTCACTCGCT
SOX9	GCTAAAGGCAACTCGTACCC	TTTCGCTGCTCCATTTAGCC
PDPN	GACCGTTTCTGACTCTGTGC	GTGAACGGTCTTTCCTTCGG
HOPX	CGCTCAACAACAACACAACG	GAGTCAGTGGAGGCGAAGAT
SFTPB	CCATACCACAGGCAATGCTC	TGCTGCTCCACAAATTGCTT
SFTPC	CCTGAGTGAGCACCTGGTTA	GCAGCTGCTGGTAGTCATAC
GAPDH	CTGACTTCAACAGCGACACCCA	GTCCACCACCCTGTTGCTGTAG

Supplementary Table 8. The primary antibodies used for western blotting

Primary antibodies	Company	Catalog number	Dilution
SARS-CoV-2 Spike Protein (S1-NTD)	Cell Signaling Technology	42172s	1:1000
SPA	Abcam	ab51891	1:1000
pro-SPC	Merck Millipore	AB3786	1:500
GAPDH (GA1R)	Thermo Scientific	MA5-15738	1:10000

Supplementary Table 9. The secondary antibodies used for western blotting

Secondary antibodies	Company	Catalog number	Dilution
HRP/IgG (H+L) goat-anti-mouse	Thermo Scientific	31430	1:5000 or 10000
HRP/IgG (H+L) goat-anti-rabbit	Thermo Scientific	31460	1:5000

Supplementary References

1. Zhang, S., Loch, A.J., Radtke, F., Egan, S.E. & Xu, K. Jagged1 is the major regulator of Notch-dependent cell fate in proximal airways. *Dev Dyn* **242**, 678-686 (2013).
2. Chung, M.I., Bujnis, M., Barkauskas, C.E., Kobayashi, Y. & Hogan, B.L.M. Niche-mediated BMP/SMAD signaling regulates lung alveolar stem cell proliferation and differentiation. *Development* **145**, dev163014 (2018).
3. Goldmann, T. et al. Human alveolar epithelial cells type II are capable of TGFβ-dependent epithelial-mesenchymal-transition and collagen-synthesis. *Respir Res* **19**, 138 (2018).
4. Yuan, T. et al. FGF10-FGFR2B Signaling Generates Basal Cells and Drives Alveolar Epithelial Regeneration by Bronchial Epithelial Stem Cells after Lung Injury. *Stem Cell Reports* **12**, 1041-1055 (2019).
5. Bove, P.F. et al. Breaking the in vitro alveolar type II cell proliferation barrier while retaining ion transport properties. *Am J Respir Cell Mol Biol* **50**, 767-776 (2014).
6. Seiji, Y. et al. Alveolus-like organoid from isolated tip epithelium of embryonic mouse lung. *Hum Cell* **32**, 103-113 (2019).
7. Nabhan, A.N., Brownfield, D.G., Harbury, P.B., Krasnow, M.A. & Desai, T.J. Single-cell Wnt signaling niches maintain stemness of alveolar type 2 cells. *Science* **359**, 1118-1123 (2018).
8. Zacharias, W.J. et al. Regeneration of the lung alveolus by an evolutionarily conserved epithelial progenitor. *Nature* **555**, 251-255 (2018).
9. Xu, H. et al. Organoid technology in disease modelling, drug development, personalized treatment and regeneration medicine. *Exp Hematol Oncol* **7**, 30 (2018).
10. Crapo, J.D., Barry, B.E., Gehr, P., Bachofen, M. & Weibel, E.R. Cell number and cell characteristics of the normal human lung. *Am Rev Respir Dis* **126**, 332-337 (1982).
11. Bradley, B.T. et al. Histopathology and ultrastructural findings of fatal COVID-19 infections in Washington State: a case series. *The Lancet* **396**, 320-332 (2020).
12. Mason, R.J. & Dobbs, L.G. Alveolar Epithelium and Pulmonary Surfactant. *Murray and Nadel's Textbook of Respiratory Medicine*, 134-149 (2016).
13. Reid, L. et al. The mysterious pulmonary brush cell: a cell in search of a function. *American journal of respiratory and critical care medicine* **172**, 136-139 (2005).
14. Overgaard, C.E., Mitchell, L.A. & Koval, M. Roles for claudins in alveolar epithelial barrier function. *Ann N Y Acad Sci* **1257**, 167-174 (2012).
15. Wu, J. et al. Characterization of air-liquid interface culture of A549 alveolar epithelial cells. *Brazilian Journal of Medical and Biological Research* **51**, e6950 (2018).
16. Ren, H., Birch, N.P. & Suresh, V. An Optimised Human Cell Culture Model for Alveolar Epithelial Transport. *PloS one* **11**, e0165225 (2016).
17. Rucka, Z. et al. Differential effects of insulin and dexamethasone on pulmonary surfactant-associated genes and proteins in A549 and H441 cells and lung tissue. *Int J Mol Med* **32**, 211-218 (2013).
18. Hermanns, M.I., Unger, R.E., Kehe, K., Peters, K. & Kirkpatrick, C.J. Lung epithelial cell lines in coculture with human pulmonary microvascular endothelial cells: development of an alveolo-capillary barrier in vitro. *Laboratory Investigation* **84**, 736-752 (2004).
19. Blanco-Melo, D. et al. Imbalanced Host Response to SARS-CoV-2 Drives Development of COVID-19. *Cell* **181**, 1036-1045 (2020).

20. Harcourt, J. et al. Severe Acute Respiratory Syndrome Coronavirus 2 from Patient with Coronavirus Disease, United States. *Emerg Infect Dis* **26**, 1266-1273 (2020).
