# Supplementary material for: Tissue-level alveolar epithelium model for recapitulating SARS-CoV-2 infection and cellular plasticity
Source: Commun Biol. 2022 Jan 19;5:70. doi: 10.1038/s42003-022-03026-3 (PMC8770515; doi:10.1038/s42003-022-03026-3)
Supplement: Supplementary file 2 — Description of Additional Supplementary Files [file 42003_2022_3026_MOESM2_ESM.pdf]

Description of Additional Supplementary Files

File name: Supplementary Data 1

Description: All data underlying the graphs and charts presented in the figures have been uploaded as a single Microsoft Excel file.

- **Fig. 1d**: EpCAM+ cells (%), Day 1 and Day 5.
- **Fig. 1e:** Vimentin+ cells (%), Day 1 and Day 5.
- **Fig. 1g:** Relative cell density (%) for each treatment (3 replicates).
- **Fig. 1h:** EpCAM+ cells (%) for each treatment (3 replicates).
- **Fig. 1i:** Vimentin+ cells (%) for each treatment (3 replicates).
- **Fig. 2d:** EpCAM+ cells (%), passages 1 – 3.
- **Fig. 2e:** Relative fold change in mRNA expression for listed genes, passages 1 – 3.
- **Fig. 3e:** Cell-density quantification of HPAEpiCs for 21 days of sub-culturing, measured on days 0, 7, 14, and 21 (3 replicates).
- **Fig. 3f:** Cell-density quantification of AT2 cells for 21 days of sub-culturing, measured on days 0, 7, 14, and 21 (3 replicates).
- **Fig. 3j:** Quantification of HTII-280^{+} cell (AT2 cell) populations for 14 days of ALI culturing, measured on days 0, 7, and 14.
- **Fig. 3k:** Quantification of phosphatidycholine concentration for 14 days of ALI culturing, measured on days 0, 7, and 14.
- **Fig. 4f:** Proinflammatory cytokine IL-6 and IL-8 concentrations produced by the HPAEpiCs after SARS-CoV-2 pseudovirus infection (MOI = 2) or mock treatment.
- **Fig. 4h:** Relative infection efficiency (%) of SARS-CoV-2 pseudovirus in HPAEpiCs following treatment with or without ACE2 antibody.
- **Fig. 5d:** Quantification of relative wound healing rate (%) of Control, CHIR99021 and XAV939 on days 0, 1, and 2 post-scratch.
