# Supplementary material for: Tissue-level alveolar epithelium model for recapitulating SARS-CoV-2 infection and cellular plasticity
Source: Commun Biol. 2022 Jan 19;5:70. doi: 10.1038/s42003-022-03026-3 (PMC8770515; doi:10.1038/s42003-022-03026-3)
Supplement: Supplementary file 4 — Reporting Summary [file 42003_2022_3026_MOESM4_ESM.pdf]

nature portfolio

Corresponding author(s): Guan-Yu Chen

Last updated by author(s): Dec 3, 2021

Reporting Summary

Nature Portfolio wishes to improve the reproducibility of the work that we publish. This form provides structure for consistency and transparency in reporting. For further information on Nature Portfolio policies, see our Editorial Policies and the Editorial Policy Checklist.

Statistics

For all statistical analyses, confirm that the following items are present in the figure legend, table legend, main text, or Methods section.

n/a	Confirmed	
☐	☒	The exact sample size (n) for each experimental group/condition, given as a discrete number and unit of measurement
☐	☒	A statement on whether measurements were taken from distinct samples or whether the same sample was measured repeatedly
☐	☒	The statistical test(s) used AND whether they are one- or two-sided<br>*Only common tests should be described solely by name; describe more complex techniques in the Methods section.*
☒	☐	A description of all covariates tested
☒	☐	A description of any assumptions or corrections, such as tests of normality and adjustment for multiple comparisons
☐	☒	A full description of the statistical parameters including central tendency (e.g. means) or other basic estimates (e.g. regression coefficient) AND variation (e.g. standard deviation) or associated estimates of uncertainty (e.g. confidence intervals)
☒	☐	For null hypothesis testing, the test statistic (e.g. F, t, r) with confidence intervals, effect sizes, degrees of freedom and P value noted<br>*Give P values as exact values whenever suitable.*
☒	☐	For Bayesian analysis, information on the choice of priors and Markov chain Monte Carlo settings
☒	☐	For hierarchical and complex designs, identification of the appropriate level for tests and full reporting of outcomes
☒	☐	Estimates of effect sizes (e.g. Cohen's d, Pearson's r), indicating how they were calculated

Our web collection on statistics for biologists contains articles on many of the points above.

Software and code

Policy information about availability of computer code

Data collection	Software used for data collection:NIS-Element Version 4.51, MetaXpress® software Version 6.5.3.427, Leica Application Suite X (LAS X) Version 3.5.7.23225, StepOne software Version 2.2.2, , Amersham Imager 600 software Version 1.2.0, and Image J Version1.53e.
Data analysis	MetaXpress® software was used to quantify microscope images acquired from the ImageXpress Micro 4 system. Image J was used to quantify the area of wound healing results. StepOne software was used to analyze gene expressions that were normalized to glyceraldehyde 3-phosphate dehydrogenase (GAPDH) housekeeping gene. All data were statistically analyzed using GraphPad Prism 8.0.2. The detailed descriptions were shown in the Method section.

For manuscripts utilizing custom algorithms or software that are central to the research but not yet described in published literature, software must be made available to editors and reviewers. We strongly encourage code deposition in a community repository (e.g. GitHub). See the Nature Portfolio guidelines for submitting code & software for further information.

Data

Policy information about availability of data

All manuscripts must include a data availability statement. This statement should provide the following information, where applicable:

- Accession codes, unique identifiers, or web links for publicly available datasets
- A description of any restrictions on data availability
- For clinical datasets or third party data, please ensure that the statement adheres to our policy

The authors declare that all other data supporting the findings of this study are available within the paper and its Supplementary information files, or are available from the corresponding author upon reasonable request.

Field-specific reporting

Please select the one below that is the best fit for your research. If you are not sure, read the appropriate sections before making your selection.

- [x] Life sciences
- [ ] Behavioural & social sciences
- [ ] Ecological, evolutionary & environmental sciences

For a reference copy of the document with all sections, see nature.com/documents/nr-reporting-summary-flat.pdf

Life sciences study design

All studies must disclose on these points even when the disclosure is negative.

Sample size	For immunofluorescent analysis, the ImageXpress Micro 4 system acquired images of all regions of cells and performed quantitative analysis. Therefore, sample size was the entire population of cells present at that time, which is the maximum possible sample size for a given experiment.
Data exclusions	Data were not excluded.
Replication	At least 3 biological replicates were performed.
Randomization	A total of 8 different donor cells were randomly assigned in the experiment. The detailed information was shown in the Supplementary Table 4.
Blinding	N/A

Reporting for specific materials, systems and methods

We require information from authors about some types of materials, experimental systems and methods used in many studies. Here, indicate whether each material, system or method listed is relevant to your study. If you are not sure if a list item applies to your research, read the appropriate section before selecting a response.

Materials & experimental systems

n/a	Involved in the study
[ ]	[x] Antibodies
[ ]	[x] Eukaryotic cell lines
[x]	[ ] Palaeontology and archaeology
[x]	[ ] Animals and other organisms
[x]	[ ] Human research participants
[x]	[ ] Clinical data
[x]	[ ] Dual use research of concern

Methods

n/a	Involved in the study
[x]	[ ] ChIP-seq
[x]	[ ] Flow cytometry
[x]	[ ] MRI-based neuroimaging

Antibodies

Antibodies used	All the information was provided in the Supplementary Tables 5, 6, 8 and 9.<br><br>The following primary antibodies were used for immunostaining and immunoblotting: α-SMA (Cell Signaling Technology, 36110S), ACE2 (Abcam, ab87436), AQP-5 (Santa Cruz Biotechnology, sc-514022 AF594), CK-18 (BioLegend, 628408), EpCAM (Cell Signaling Technology, 5447S), EpCAM (BioLegend, 324214), HTI-56 (Terrace Biotech, HT1-56), HTII-280 (Terrace Biotech, HT2-280), PDPN (Abcam, ab10288), pro-SPC (Merck Millipore, AB3786), SOX9 (Abcam, ab196184), SPA (Abcam, ab51891), SPB (Thermo Scientific, MA1-204), SPD (Bioss, BS-1583R), TMPRSS2 (Santa Cruz Biotechnology, sc-515727), Vimentin (Cell Signaling Technology, 9854S), ZO-1 (Thermo Scientific, 339188), ZO-1 (Cell Signaling Technology, 13663S), SARS-CoV-2 Spike Protein (S1-NTD) (Cell Signaling Technology, 42172s), and GAPDH (GA1R) (Thermo Scientific, MA5-15738).<br><br>The following secondary antibodies were used for immunostaining and immunoblotting: Alexa Fluor® 488/IgG (Jackson ImmunoResearch, 115-545-062), Alexa Fluor® 488/IgG (Jackson ImmunoResearch, 111-545-003), Alexa Fluor® 488/IgM (Thermo Scientific, A-21042), Alexa Fluor® 555/IgG1 (Thermo Scientific, A-21127), Alexa Fluor® 633/IgM (Thermo Scientific, A-21046), Alexa Fluor® 647/IgG (Jackson ImmunoResearch, 115-605-003), Cy3/IgG (Merck Millipore, AP132C), HRP/IgG (H+L) goat-anti-mouse (Thermo Scientific, 31430), and HRP/IgG (H+L) goat-anti-rabbit (Thermo Scientific, 31460).
Validation	All the antibodies used are commercially available and have been validated on manufacture's website or published papers.

Eukaryotic cell lines

Policy information about cell lines

Cell line source(s)	Human pulmonary alveolar epithelial cells (HPAEpiCs) were purchased from ScienCell Research Laboratories (ScienCell, 3200).
Authentication	The HPAEpiCs have been authenticated by the manufacturer and their related phenotypes have also been analyzed in the paper.
Mycoplasma contamination	The HPAEpiCs were test negative for HIV-1, HBV, HCV, mycoplasma, bacteria, yeast and fungi.
Commonly misidentified lines (See ICLAC register)	N/A
